# Movement Dependence and Layer Specificity of Entorhinal Phase Precession in Two-Dimensional Environments

**DOI:** 10.1371/journal.pone.0100638

**Published:** 2014-06-24

**Authors:** Eric Reifenstein, Martin Stemmler, Andreas V. M. Herz, Richard Kempter, Susanne Schreiber

**Affiliations:** 1 Institute for Theoretical Biology, Department of Biology, Humboldt-Universität zu Berlin, Berlin, Germany and Bernstein Center for Computational Neuroscience Berlin, Berlin, Germany; 2 Department Biology II, Ludwig-Maximilians-Universität München, Planegg-Martinsried, Germany; and Bernstein Center for Computational Neuroscience Munich, Munich, Germany; McGill University, Canada

## Abstract

As a rat moves, grid cells in its entorhinal cortex (EC) discharge at multiple locations of the external world, and the firing fields of each grid cell span a hexagonal lattice. For movements on linear tracks, spikes tend to occur at successively earlier phases of the theta-band filtered local field potential during the traversal of a firing field – a phenomenon termed phase precession. The complex movement patterns observed in two-dimensional (2D) open-field environments may fundamentally alter phase precession. To study this question at the behaviorally relevant single-run level, we analyzed EC spike patterns as a function of the distance traveled by the rat along each trajectory. This analysis revealed that cells across all EC layers fire spikes that phase-precess; indeed, the rate and extent of phase precession were the same, only the correlation between spike phase and path length was weaker in EC layer III. Both slope and correlation of phase precession were surprisingly similar on linear tracks and in 2D open-field environments despite strong differences in the movement statistics, including running speed. While the phase-precession slope did not correlate with the average running speed, it did depend on specific properties of the animal's path. The longer a curving path through a grid-field in a 2D environment, the shallower was the rate of phase precession, while runs that grazed a grid field tangentially led to a steeper phase-precession slope than runs through the field center. Oscillatory interference models for grid cells do not reproduce the observed phenomena.

## Introduction

Large-scale oscillations can organize the spikes of individual neurons [1]. In some cases, neural discharges are precisely orchestrated such that spike phases relative to an ongoing oscillation of the local field potential (LFP) convey information about a visual scene, the identity of a memory item, or the location of an animal [2]–[4]. The entorhinal-hippocampal complex in rodents, for instance, exhibits prominent LFP oscillations in the theta band (6–11 Hz) when the animal explores its environment. For certain neurons within this complex, the theta-band spike phase decreases with distance traveled through the neuron's firing field, a phenomenon known as phase precession [5].

Many neurons in the entorhinal-hippocampal complex are spatially tuned. Grid cells in the medial entorhinal cortex (mEC) form some of the most elaborate spatial firing rate maps known – multiple receptive fields arranged in a hexagonal grid [6]. Hippocampal place cells, in contrast, often have only a single firing field in a given environment, although firing fields do repeat under certain conditions [7]–[10].

On linear tracks, grid cells show phase precession [11], just as place cells do. In two-dimensional environments, the spikes of place cells as well as grid cells precess in pooled-run data [12]–[14], but it is unknown whether the same is true for individual field traversals. Single-run phase precession has been shown for linear-track data from hippocampal place cells [15]. For entorhinal grid cells, the spacing and size of firing fields differs between one- and two-dimensional environments [11], [16]. Moreover, a rat's behavior changes within these two environments: on a linear track, the animal runs in a stereotyped, goal-directed manner, while foraging in a two-dimensional environment, the animal's trajectories and its running speed are highly variable. Paths can curve, go through the center of the grid field, or swerve and miss it completely; the time spent in the grid field varies as the rat slows down or speeds up.

These factors might severely change or even obscure the signatures of grid-cell phase precession. Therefore, we examined phase-precession on a run-by-run basis in two-dimensional environments – a strategy previously applied to linear-track data [15], [17], [18]. We first evaluated phase-precession properties in dependence upon the properties of the two-dimensional path. Because cells in different mEC layers differ in their preferred spike phases [17], we also investigated the layer specificity of phase precession at the single-run level.

The results of our data analysis provide additional constraints for computational models. One class of model relies on a baseline theta oscillation and additional oscillators whose frequencies increase linearly with speed along certain preferred directions [19], [20]. As the carrier frequency of the resulting beat pattern is higher than the baseline frequency, spikes will precess relative to this baseline. Other models explain grid fields through attractor dynamics [21]–[23]. Yet attractor networks do not intrinsically explain phase precession, but require additional mechanisms, such as after-spike dynamics [24] or oscillatory interference [22]. An intracellular ramp depolarization poses another common explanation for phase precession [25]. However, it does not explain phase precession at the edges of a firing field. Therefore, we focus on different versions of the oscillatory interference model and ask whether they can reproduce single-run phase precession as in the experimental data.

## Materials and Methods

We reanalyzed – both published and previously unpublished – data that were recorded by Hafting et al. [11] and Sargolini et al. [26]. In these experiments, extracellular recordings were performed in the medial entorhinal cortex (mEC) of 7 rats that explored a 1 m^2^ square box [26] or ran on a linear track [11]. Data include sorted single-unit activity, the local field potential (LFP) sampled at 250 Hz and recorded from the same electrode as the spiking activity (low-pass filtered at 500 Hz, single pole), and the position of the rat, which was tracked by a diode fixed to the animal's head. The data recorded by Hafting et al. [11] and Sargolini et al. [26] are available at http://www.ntnu.no/cbm/moser/gridcell.

For our analysis, spikes were partitioned into firing fields, and each spike was assigned spatial coordinates, convolving these coordinates with a Gaussian kernel of width 5 cm and dividing by the time spent at each location resulted in a firing-rate map. The borders of a candidate firing field were obtained by initially thresholding the firing-rate map at 20% of the overall peak rate (bin size: 1 cm×1 cm). Those borders were then further extended to 20% of the individual firing field's peak rate. Fields with an area of less than 200 cm^2^ or a circumference of more than 160 cm were excluded from the analysis (74 out of 388); the remaining grid fields were almost circular. We also tested different values for the initial firing-rate threshold (see Fig. S1 in File S1), which led to either smaller fields (threshold of 25%) or even merged fields (low threshold values like 5% or 10%). We were able to replicate all of our findings for a threshold value of 15%.

For each cell the gridness score [27] was calculated. Cells with a gridness score of less than 0 were excluded from the analysis. Eighty-seven units with a total of 314 grid fields were analyzed. As the borders of the environment limit an animal's movements, we separately analyzed the dependence of phase precession on the properties of the rat's path by considering the 115 grid fields that had no overlap with the boundaries of the box; we confirmed that the results presented for all 314 grid fields were not affected by the overlap of fields with the boundaries. The average area of the 115 central fields was 509 cm^2^, corresponding to an average field diameter of 24.9 cm if one assumes a circular field shape.

**Figure 1:**
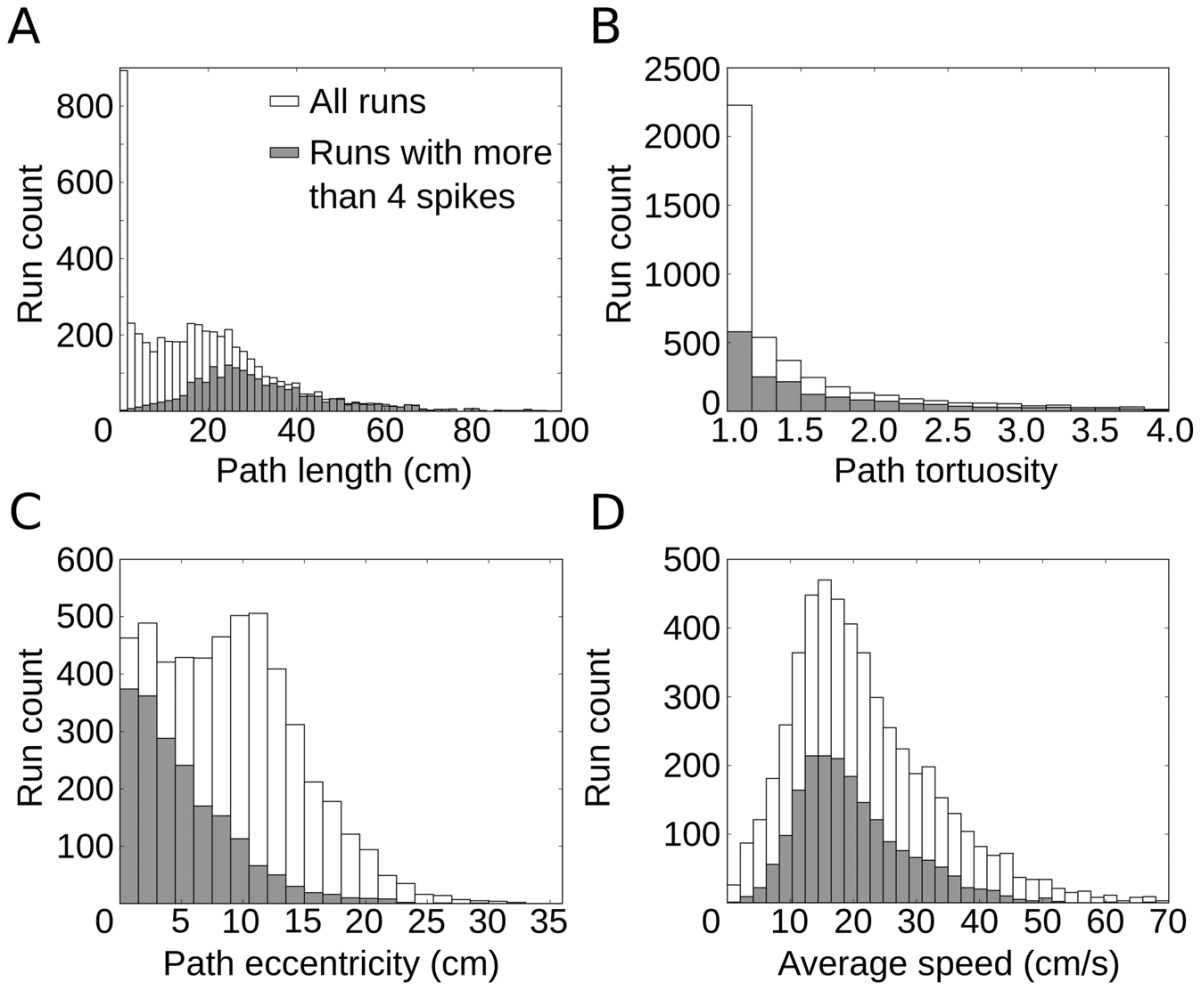
Statistical properties of runs through single grid fields. (A) Distribution of path length within grid fields for all runs and for all runs with more than four spikes. In many runs, the animal's paths graze the field. Most of these very short runs show only little spiking activity. (B) Distribution of path tortuosity, as measured by the ratio of the actual path length to the length of the straight line connecting field entry to field exit. Short runs from (A) are usually straight. (C) Distribution of path eccentricity, the shortest distance between the path and the location of the maximum firing rate within the field (the “firing-field peak”). Note that many runs with large eccentricities have only few spikes. (D) Distribution of average speed in single runs. The constraint on spiking activity only mildly affects this distribution.

All told, there were 7139 single-firing-field crossings, or “runs”; on 4396 of these, the cell fired spikes. For each run, four properties were assessed: (1) path length, measured along the animal's trajectory from entry to exit of the firing field; (2) path tortuosity, the ratio of the actual path length to the length of the straight line connecting entry to exit; (3) path eccentricity, measured as the shortest distance between the path and the location of the maximum firing rate within the field (the “firing-field peak”), and (4) average speed within the firing field. The statistics of these run properties are shown in Figure 1. Note that many runs were short, straight, and tangential. These runs usually showed only little spiking activity. Runs with a tortuosity of <1.4 were considered “straight runs”. We also checked that our main results still hold if only runs were taken that lasted at least 3 theta cycles and on which the rat did not move slower than 1 cm/s at any time during field traversal (see Figures S3, S4 and S5 in File S1).

The LFP signal was band-pass filtered in the theta frequency range (6–11 Hz). Every spike was assigned an instantaneous theta phase, using the Hilbert transform of the filtered signal. This procedure sets phase 0° to the ascending slope of the oscillation; the peak hence is associated with a phase of 90°. In contrast, Hafting et al. [11] defined the peak of the oscillation as phase 0°. Phase precession was quantified by two measures [15], [18], [28]: first, the slope *m* from circular-linear regression, which results from fitting the model 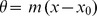 to the data (where 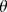 is the phase, 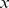 is the spatial variable and 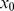 the spatial offset) by maximizing 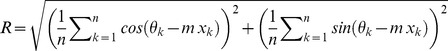
and, second, the circular-linear correlation coefficient



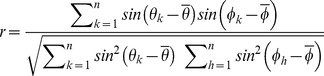
where 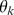 denotes the theta phase of the k-th spike (n spikes in total), and 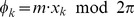 is a circular variable that is derived from the animal's position 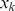. The phases 
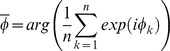
 and 
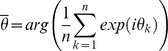
 are the circular sample mean values. The significance value *p* (null hypothesis: *r = 0*) can be calculated as 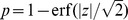, where 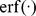 depicts the error function and 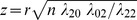 with 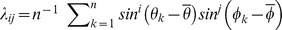 [28], [29].

Due to the circular nature of the phase variable, fitting the phase-precession slope *m* is often ambiguous for low spike numbers. The analysis was therefore performed on runs with more than four spikes (n = 2466). Results were similar if this criterion was changed to require more than 3, 5 or 6 spikes per run (Fig. S6 in File S1). To avoid overfitting the data by a helix that spirals around the phase-position cylinder many times, we restricted slopes to [−60,60] deg/cm. Restrictions to [−50,50] and [−80,80] deg/cm gave similar results (Fig. S7 in File S1).

**Figure 2:**
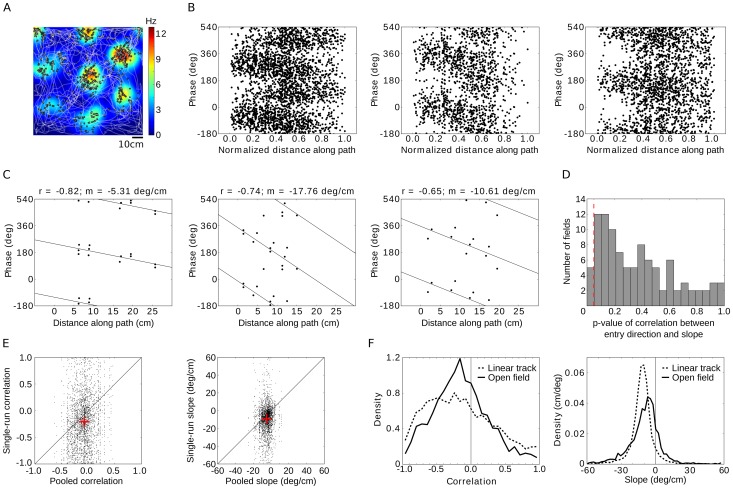
Grid cells exhibit phase precession in two-dimensional environments. (A) Trajectory (white line) of a rat over 10 minutes in a 1 m^2^ square enclosure together with the firing pattern (black dots) and color-coded firing-rate map of a single grid cell. Data from Sargolini et al. [26]. Note that many different paths traverse each grid field. (B) Spike phase relative to the local field potential for all passages through three example grid fields. Runs with varying directions and originating from various points in the two-dimensional environment are pooled. The position along each path within the firing field is normalized by the path's total length. (C) Three examples of single runs with different phase-precession slopes *m* and circular-linear correlation values *r*. Circular-linear regression lines are indicated. (D) Running direction has no consistent influence on the phase-precession slope. Histogram of p-values of the correlation between entry direction of the animal into a firing field and single-run phase-precession slope. The analysis is restricted to straight runs. Red dashed line indicates significance level p = 0.05. (E) Comparison of single-run phase precession and phase precession assessed by pooling all runs through a particular grid field. Each dot represents a single run; the left panel shows the place-phase correlation, the right panel depicts the slope of phase versus location. A negative slope implies phase precession; note the large variability across different runs. Red crosses denote the average correlation and the average slope. The diagonal line marks the identity. (F) Single-run phase precession in one and two-dimensional environments. (*left*) Distribution of circular-linear correlation values for runs on a linear track (dashed lines) and in the square arena (full lines). (*right*) Distribution of phase-precession slopes for the same two conditions. Despite the large speed and movement differences between the linear track and the open field, the phase-precession statistics are similar.

To assess directional influences on phase precession, we measured the linear-circular correlation [30], [31] between entry direction to the firing field and phase-precession slope on a field-by-field basis. Note that this measure is different from the circular-linear correlation used throughout the manuscript: for the analysis shown in Fig. 2D, the angular variable is the independent variable, whereas in the remaining manuscript the angular variable is the dependent variable. The linear-circular correlation takes values between 0 and 1 and allows for the calculation of a p-value. P-values below 0.05 were used to indicate a statistically significant correlation. To reliably measure the correlation and p-value, only fields that contributed more than four runs were included in this analysis.

To test for significance of a difference between mean values of two data samples, we generally used two-sample two-tailed t-tests. As correlation coefficients turned out to stem from strongly skewed distributions, the Wilcoxon rank-sum test – assessing whether the medians of two sets of sampled data are the same – was used to analyze correlations. To compare the means of multiple groups, we used one-way ANOVA. Moreover, to disentangle the influences of path length, tortuosity, eccentricity, and speed on the phase-precession slope, a 4-way ANOVA without interaction terms was used. P-values were obtained by comparing the full model to a reduced model without the factor in question; e.g. for path length, the full model (4 parameters) was compared to a model that contained only tortuosity, eccentricity, and speed.

We used the Rayleigh test to test for circular uniformity. Additionally, we used the vector strength to quantify theta phase preference. The p-value indicates the likelihood of observing a result that is as least as extreme as the one that was actually observed, assuming that the null hypothesis (“equal means”, “equal medians” or “data is uniform”, respectively) is true. The null hypothesis was rejected when p<0.05.

Three variants of the oscillatory interference model were implemented [19], [32]. In the first two models, a voltage-like variable 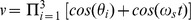 results from the threefold superposition of cosines, with 
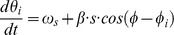
, where 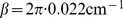 and 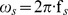 with 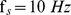. The parameters 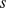 and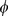 are the speed and direction of the animal's movement. The preferred directions 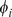 of the three oscillators were chosen to be 60° apart for the first version and 120° apart for the second version of the model.

In the third model of oscillatory interference, three pairs of oscillators were used, for a total of six. By imposing half-wave rectification, the frequency of each oscillator never falls below the theta frequency 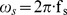 with 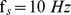. The equation for the voltage-like variable reads 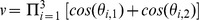 with 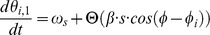 and 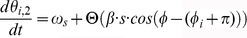. Here, 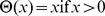, but is zero otherwise. The three preferred directions were 0°, 60°, and 120°.

In all versions of the model, spikes were produced by applying a threshold to the voltage variable. Spikes phases were assigned with respect to the baseline oscillation at an angular frequency 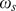. Thresholds were chosen to approximate grid-field sizes in the experimental data. In the first two models 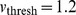, while the third model had 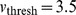.

## Results

To answer key open questions about phase precession in rat entorhinal cortex, we analyzed in-vivo data recorded in freely moving animals in 1D and 2D environments on the basis of individual runs. In contrast to estimates that consider data pooled across multiple runs or firing fields [5], [11]–[14], [17], [33], the single-run approach enabled us to directly relate phase precession to the properties of the animal's paths through a cell's spatial firing field. Moreover, we addressed how phase precession depends on the anatomical layer of the spiking cell for pooled and single-run estimates of phase precession. Complementing this approach, we used the single-run analysis to test whether the observed features of phase precession were reproduced by models that explain both the formation of the spatial firing-rate map for grid cells and their phase precession as the result of multiple oscillations at different frequencies.

### Phase precession prevails in single runs through 2D environments

Extracellularly isolated cells in the medial entorhinal cortex (mEC) and the corresponding local field potential (LFP) were recorded by Sargolini et al. [26] in rats that explored a square arena (1 m^2^) while searching for food; the data were made available by E.I. Moser (Norwegian University of Science and Technology, Trondheim, Norway). Eighty-seven recorded cells showed spatial firing maps with hexagonal grid structure, resulting in a total of 314 clearly discernible firing fields (see also Materials and Methods). The paths taken through these grid fields varied widely (Fig. 2A). We tested how the phase of grid-cell firing changes along a rat's path within a firing field, as measured by the distance traveled. We found that grid cells fired at earlier phases as the animal moved along its path. Three typical examples of such phase-precession patterns, pooled over all traversals (or “runs”) through a given firing field, are shown in Fig. 2B. For visualization of the pooled data, the traveled distance was normalized by the total path length in each run. Throughout the manuscript, all analyses were performed using the absolute traveled distance along the path (in cm). Phase precession also occurred at the level of single runs, as demonstrated by three sample runs from different cells and animals with clearly negative phase-precession slopes (Fig. 2C).

The running direction at field entry spanned 360°. Yet, running direction had no consistent influence on the phase-precession slope (Fig. 2D). We measured the linear-circular correlation coefficient between the running direction at field entry and the slope on a field-by-field basis (see also Materials and Methods). The resulting distribution of p-values of the linear-circular correlation is shown for straight runs (tortuosity <1.4). Only 5 out of 100 fields showed a significant correlation between entry direction and slope, as expected by the statistical significance criterion of 5%. Similarly, when all runs (straight and curved) were included in the analysis, entry direction and slope were significantly correlated in only 4 out 190 fields (about 2%). For those analyses, fields were required to have more than 4 runs – straight runs or all runs, respectively.

Despite the propensity of grid-cell spikes to precess on single runs, the properties of phase precession varied greatly from run to run. The resulting correlation coefficients between phase and distance traveled along the path (Fig. 2E, left panel) and phase-precession slopes (Fig. 2E, right panel) were broadly distributed. Nevertheless, single-run phase precession turned out to be more tightly correlated and steeper than pooled phase precession (Fig. 2E, Wilcoxon rank-sum test p<10^−8^ for the correlation, t-test p<10^−8^ for the slope). At the single-run level, the median circular-linear correlation was r = −0.17 (95% confidence interval [−0.19, −0.15]) and the phase precessed by −8.4±0.3 deg/cm on average. Figure 2F compares the phase precession in 1D and in 2D environments, again at the single-run level [11], [18]. The correlations on the linear track were stronger, r = −0.20 (95% confidence interval [−0.22, −0.19]), and the slope of the relation between phase and position (−10.2±0.1 deg/cm) was about 20% steeper than in the square enclosure. We also found that the firing rate influences the phase-precession correlation, but has no effect on the slope (see Fig. S8 in File S1 for details).

### Dependence of phase precession on specific path features

**Figure 3:**
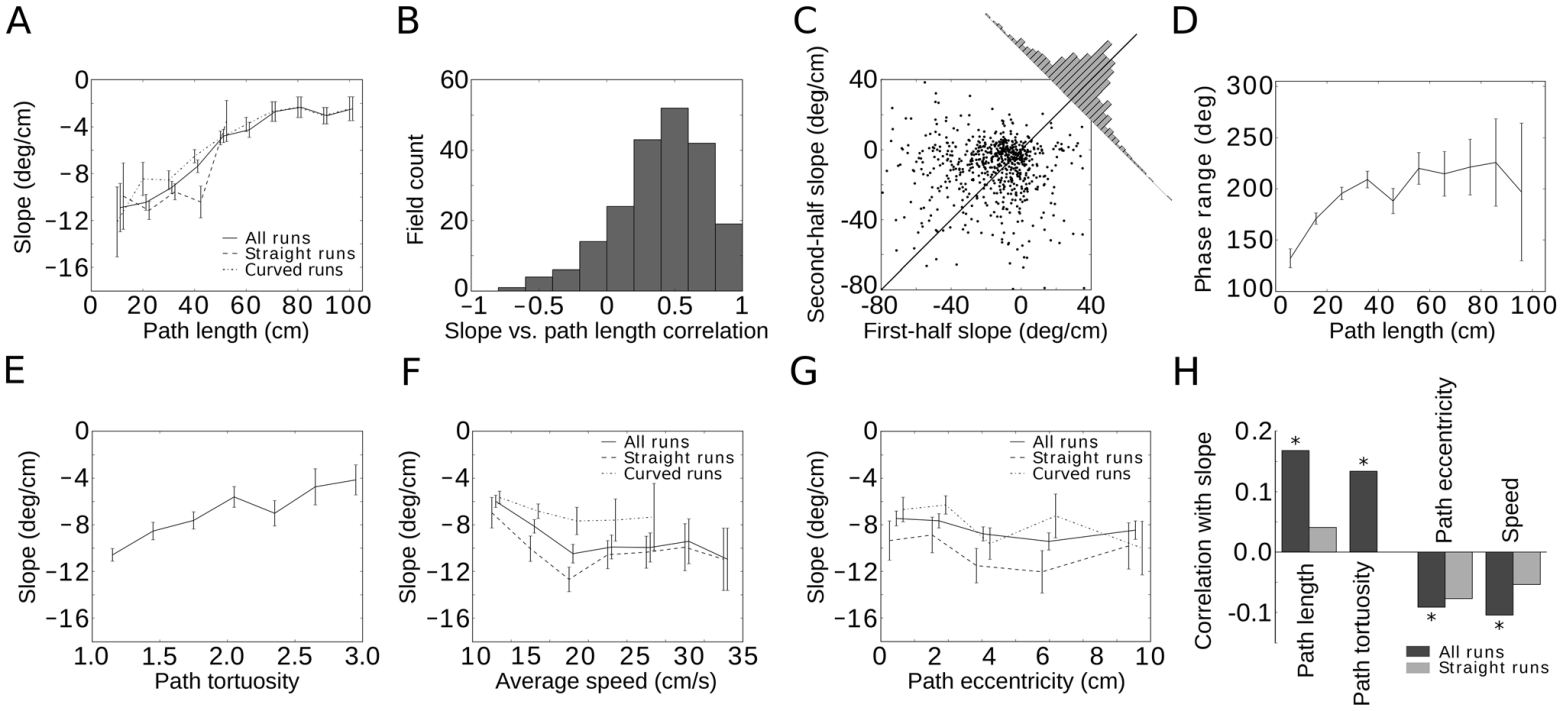
Salient features of the animal's path through a grid field affect phase precession. (A) The shorter the path is, the steeper the phase precession becomes. (B) The path length and phase precession correlate on a grid field by grid field basis, not just on average across grid fields. (C) First-half slopes are steeper than second-half slopes. The histogram in the inset shows the distance of data points from the diagonal, which is skewed towards smaller slopes in the second half of runs. (D) The phase range increases with path length and saturates at about 210°. (E) More meandering runs (increasing tortuosity) exhibit a less pronounced phase precession. As tortuosity correlates with the path length in a firing field, this finding is consistent with (A). (F) The animal's speed affects the phase-precession slope only weakly, and this effect primarily reflects a correlation between speed and tortuosity. For straight runs through the field, a statistically significant effect of speed on phase precession was not found. (G) Tangential paths lead to steeper phase precession than paths through the center of the field. The eccentricity measures the shortest distance between the path and the center of the firing field. For straight runs, the effect is not statistically significant. (H) Summary of the observed phenomena, with asterisks indicating statistical significance (p<0.05). For all investigated measures, restricting the analysis to straight runs weakens the effects. Error bars indicate one s.e.m. and are slightly offset for clarity in (A), (F), and (G).

Rather different paths can be taken through a grid field – long or short, curved or straight, fast or slow (see also Fig. 1). Therefore, we investigated whether phase precession depended on the properties of the path taken. We found that the longer the path, the shallower the slope of phase precession became (Fig. 3A, Pearson correlation r = 0.17, p<10^−12^; see also Fig. 3H). Fields with smaller diameters were generally associated with steeper phase-precession slopes (Pearson correlation r = 0.35, p<10^−8^), but even within a single field of fixed size, longer paths were associated with shallower slopes of phase precession. Overall, the majority of firing fields (across all cells) showed a positive correlation between phase-precession slope and path length (Fig. 3B), in agreement with Fig. 3A. Measuring the phase-precession slopes during the first and second half of long runs (>60 cm) revealed that the slope during the second half was significantly shallower than during the first half (Fig. 3C, p<10^−5^, t-test). The total phase shift during phase precession, measured as precession slope times run distance, was substantial even for short runs with relatively few spikes (∼150°). For longer paths, the phase range first increased and then approached a value of about 210° (Fig. 3D; average range for runs longer than 50 cm: 211°±9°). Thus phase precession started off more steeply and flattened out later in a run through a grid field.

The rat's path in a 2D environment need not be straight. In general, long runs resulted from winding trajectories through a firing field. To quantify the influence of curving paths on phase precession, we defined the tortuosity of a path as the distance along that path divided by the length of the straight line between field entry and exit of the path in question. We found that phase precession became shallower as a function of the path's tortuosity (Fig. 3E, r = 0.13, p<10^−7^), as it did for longer paths (Fig. 3A). Moreover, rats ran more slowly on meandering paths (r = −0.39, p<10^−10^), so that slower speeds tended to result in slower rates of phase precession (Fig. 3F, r = −0.10, p = 10^−5^). When we restricted the analysis to straight runs (Materials and Methods), however, we found no significant correlation between speed and slope (Fig. 3F, r = −0.05, p = 0.14).

In contrast to data from 1D environments, paths in 2D allowed us to directly address the question whether phase precession differs between runs that pass through the center of a field and those that only skirt the grid field tangentially. We defined the eccentricity of a path as the shortest Euclidean distance between the path and the center of the firing field. Slope and eccentricity correlated negatively for all runs (Fig. 3G, r = −0.09, p<0.04): the more a run “grazed” the firing field, the steeper the phase precession slope. The distance between field entry and exit was shorter on tangential runs (correlation between path length and eccentricity r = −0.31, p<10^−10^), consistent with the analysis of pooled runs by Jeewajee et al. [13] and Climer et al. [14], and shorter runs generally showed a steeper slope of phase precession (Fig. 3A). Restricting the analysis to straight runs weakened the correlation between slope and eccentricity, path length, and speed (Fig. 3H).

Based on the correlation and p-values, path length and tortuosity seemed to be the most important factors. To investigate this question more closely, we performed an analysis of variance on the effect of path length, tortuosity, eccentricity, and speed on the slope, quantifying how much each of these four components contributes to explaining the slope value (see Methods). We found that path length (p<10^−5^) and tortuosity (p = 0.02) significantly improved the model's performance, whereas eccentricity (p = 0.05) and speed (p = 0.31) did not. In summary, phase precession depended on the properties of a path through the firing field, and path length and path tortuosity were the most important factors.

**Figure 4:**
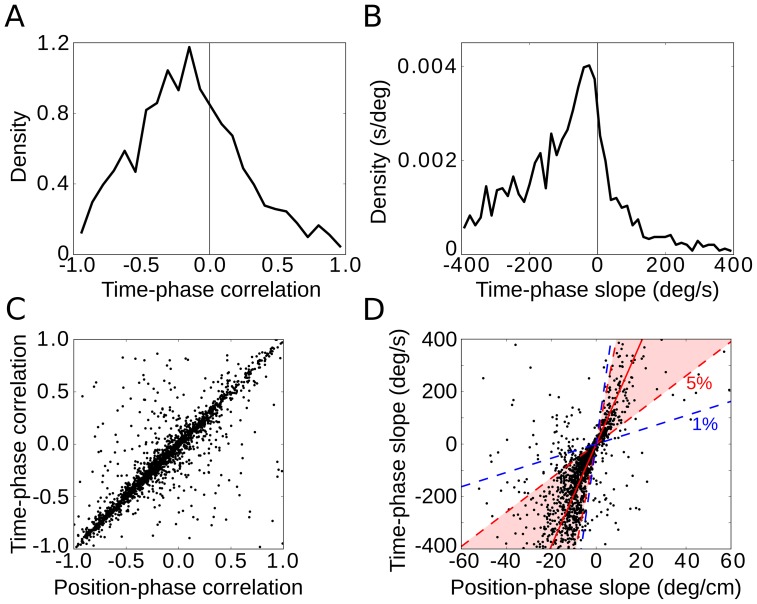
Phase precession in time. Negative values of time-phase correlation (A) and time-phase slope (B) of single runs indicate phase precession. (C) Time-phase correlation and position-phase correlation are statistically indistinguishable in single runs. (D) Time-phase slope and position-phase slope are highly correlated. The slope of the solid red line indicates the median of average speeds (19.5 cm/s) in single runs. Red and blue dashed lines mark 5-percentiles (6.5 cm/s and 44.1 cm/s, region shown in magenta) and 1-percentiles (2.7 cm/s and 59.9 cm/s) of the speed distribution, respectively. These data show that the variability between the phase-time and phase-position slope is mainly due to variations of the animals' running speed.

In hippocampal place cells recorded in rats running on a linear track, the correlation of spike phase with spatial location on a run-by-run basis is as strong as the correlation with time [15]. Whether the same is true for grid cells in 2D environments is not obvious, as time and Euclidean distance traveled do not correlate nearly as strongly when the rat has more freedom to move. Hence we next measured the correlation and slope of the spike phases with respect to time elapsed since field entry. We found the median correlation and the mean slope to be clearly negative (median time-phase correlation: −0.17, mean time-phase slope: −86.9 deg/s, see Fig. 4A, B). Interestingly, the median of time-phase correlations was statistically indistinguishable from the median of position-phase correlations in single runs (Fig. 4C, Wilcoxon rank-sum test p = 0.44). Time-phase slope and position-phase slope were strongly correlated (r = 0.65, p<10^−10^, Fig. 4D). Indeed, much of the variability between the phase-time and phase-position slope could be explained by variations in speed. Within the scatter graph of time-phase slope (in deg/s) plotted against position-phase slope (in deg/cm, see Fig. 4D), any straight line through the origin corresponds to a constant speed, with units of (deg/s)/(deg/cm)  =  cm/s. The 5-percentile lines bound a cone in which 90% of the rat's speeds fell, covering 6.5 cm/s to 44.1 cm/s; 82.0% of the scatter plot's points were found within this region. At the 1-percentile level, for speeds ranging from 2.7 cm/s and 59.9 cm/s, 90.1% of the points fell within the corresponding cone. We also tested the path-dependence of phase precession with respect to the phase-time slope and confirmed the effects of path length, tortuosity and eccentricity (Fig. S9 in File S1). The speed of the animal was negatively correlated with the phase-time slope. This result is consistent with the lack of a speed-effect on the phase-position slope for straight runs.

### Layer specificity of EC phase precession

**Figure 5:**
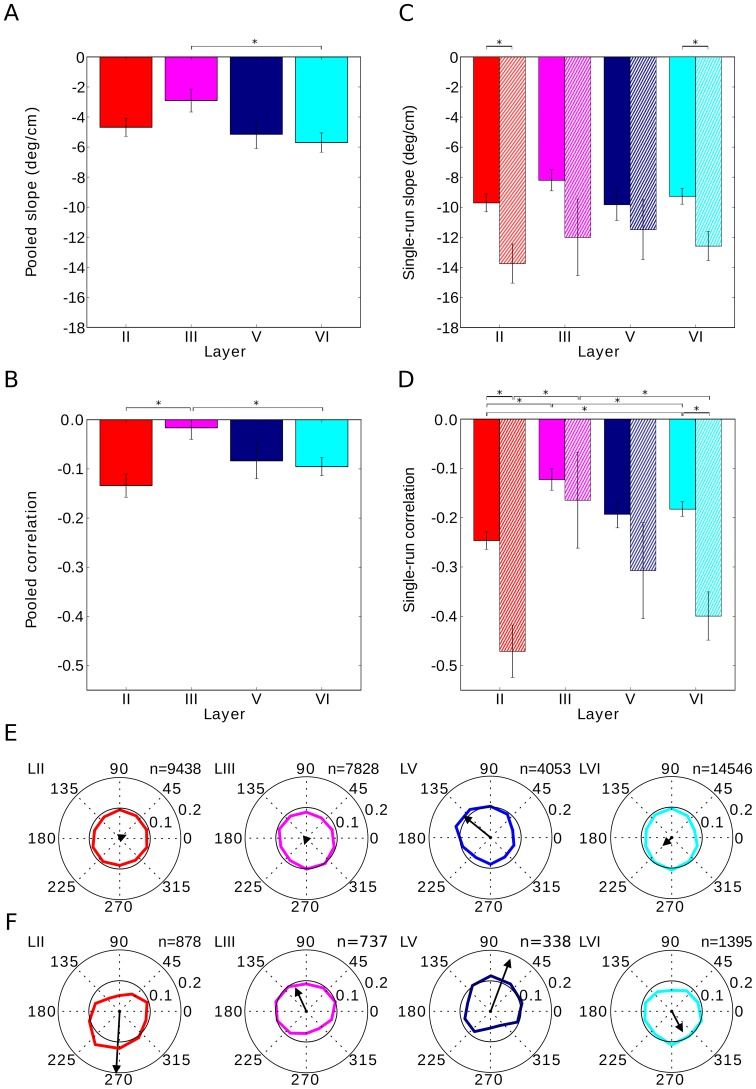
Phase precession in different cortical layers. Phase-precession slope generally does not depend on the cell's cortical layer (A and C). However, phase precession is decreased in layer III, as measured by the correlation (B and D). The single-run correlation of phase precession is lowest in layer III, and in layers II, V and VI the place-phase correlation is similar. Single-run effects are reproduced when the analysis is restricted to significantly correlated runs (cross-hatched bars). All bars show mean values, error bars depict one s.e.m. and asterisks indicate statistical significance (p<0.05). (E) Spikes show a preferred theta phase. The theta-phase preference is mild, and the weakest phase locking is encountered in layer III. (F) The first spike in a grid-field traversal generally occurs late in the theta cycle for layer II and VI, while it occurs rather early in layers III and V. In (E) and (F), the spike count histogram is normalized so that the sum of all ten bins equals 1. Colors label the cortical layer. Black arrows indicate the vector strength of the spike-phase theta modulation. All spikes were included in the analysis; no prior selection was made. The analysis is based on a total of 95 cells: 20 cells for layer II, 36 from layer III, 10 from layer V and 29 from layer VI.

Earlier studies [11], [17] suggested that phase precession is prominent in cortical layer II but weak in layer III. We reanalyzed this question by pooling across fields and runs. Although slopes of phase precession did not show significant differences across cortical layers (Fig. 5A, note the one exception), we confirmed that phase-precession correlations depend on the cell's layer in mEC (Fig. 5B). Spikes from layer II cells clearly precessed, while deep layers displayed a slightly weaker correlation between phase and the animal's position, and layer III showed hardly any correlation (Fig. 5B).

We then turned to the analysis of single runs and found that, on a run-by-run basis, phase-precession slopes did not show a statistical difference across different cortical layers (Fig. 5C, one-way ANOVA, p = 0.35). On the other hand, the correlation of phase precession was clearly smaller in layer III (t-test layer II versus layer III: p<10^−5^, Fig. 5D, but note the differential effects for conjunctive cells and pure grid cells, Fig. S14 in File S1) – the phases were much more variable. Single-run phase precession in the deep layers of mEC was comparable to phase precession in layer II (t-test correlation in layer II versus layer V: p = 0.12, correlation II versus VI: p = 0.006 slope II versus V: p = 0.92, slope II versus VI: p = 0.59); no difference could be discerned between layers V and VI (t-test correlation in layer V versus VI: p = 0.76, slope V versus VI: p = 0.65). When we restricted the single-run analysis to traversals with a significant correlation between position and phase (p<0.05), we could confirm the findings described above: phase-precession slopes did not depend on cortical layer (one-way ANOVA, p = 0.78), whereas layer III showed a lower phase-precession correlation than layers II and VI (t-test of correlations in layer II versus layer III: p = 0.003, II vs. V: p = 0.14, II vs. VI: p = 0.33, III vs. V: p = 0.35, III vs. VI: p = 0.02, V vs. VI: p = 0.40).

Independently of the strength of phase precession, spikes may preferentially occur at a particular phase of the theta rhythm. The theta phase preference was not pronounced, though, and differed mildly from layer to layer, being strongest in layer V and weakest in layer II (Fig. 5E, vector strengths: layer II: 0.02, layer III: 0.02, layer V: 0.11, layer VI: 0.03; Rayleigh-test for circular uniformity: layer II: p = 0.63, layer III: p = 4*10–6, layer V: p<10–10; layer VI: p<2*10–7). Furthermore, phase precession implies that the first spike in a firing field should occur at a different phase than the “average” spike. Cells in layer II displayed a shift of about 220° between the first spike and the median spike (Fig. 5F), with the first spike occurring late in the theta cycle.

### Comparison with oscillatory interference models

**Figure 6:**
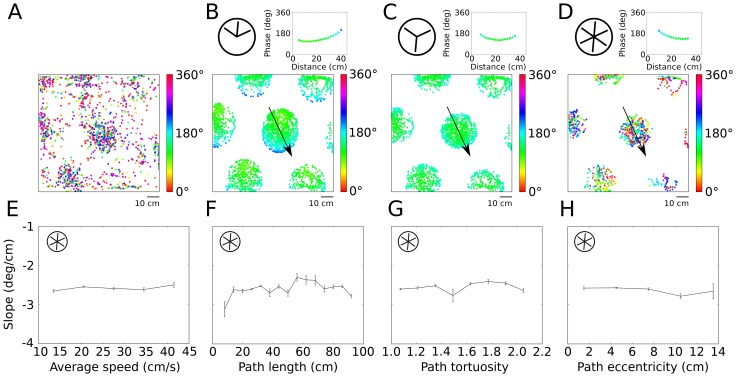
Testing predictions of oscillatory interference models. (A) Spikes (dots) of an example grid cell in a two-dimensional environment. Colors indicate the theta phase of spiking. (B) Oscillatory interference model with three “dendritic” oscillations; preferred directions are separated by 60°, as indicated in the inset at the top left, yields direction-dependent phase coding. The top right inset shows spike phases along a linear run through the central firing field as indicated by the arrow in the main panel. (C) Preferred directions separated by 120° lead to nonmonotonic phase coding so that spike phases first precess, then recess. Insets show phases for linear runs through the center, as in (B). (D) Model with six “dendritic” oscillators whose frequency modulation with speed is half-wave rectified such that the frequency never falls below theta frequency. This model leads to saturating phase precession for any run through a grid field, but the phase-precession slope is independent of path length (E), tortuosity (F), and eccentricity (G), which is in contrast to the data analysis in Figure 3. (H) The rate of phase precession does not depend on speed, which is consistent with the experimental data for straight runs.

The observed properties of phase precession in 2D environments can serve to constrain computational models of phase precession in grid cells. As a last step of our analysis, we therefore asked whether the interference of oscillations at different frequencies [19], [20], [32] can explain the experimental data in two-dimensional arenas. We investigated different versions of oscillatory interference models for grid-cell formation (see Material and Methods) and compared the models to the in-vivo data (Fig. 6A). The original oscillatory interference model uses three speed- and head-direction-dependent oscillators and one reference oscillator that represents the theta rhythm. When the animal moves, the frequency of the oscillator aligned to the running direction increases relative to the reference frequency, which causes an interference pattern that oscillates at half of the frequency difference. A grid pattern can be formed by three speed-controlled oscillators with different preferred running directions. In a simple version of the model, the input oscillators have preferred directions that are 60° apart. This model leads to direction-dependent phase coding (also see [14]); Fig. 6B depicts an extreme case in which phase-recession (and not phase-precession) results along a particular direction. Such behavior was not observed in the in-vivo data. In fact, our data showed that phase precession occurs independently of heading direction of the rat in two dimensions.

A second version of the model uses three input oscillators whose preferred directions are separated by 120°. This model leads to phase coding that is independent of running direction. For this angular separation of oscillators, the sinusoidal modulation of frequency with heading direction implies that at least one oscillator's frequency falls below the reference frequency – for any direction of motion. The input oscillators' frequencies, therefore, fall both below and above the reference frequency, the phase changes non-monotonically with position (Fig. 6C). For this specific model, the phase first precesses and later recesses.

An extended version of the original model is based on six “dendritic” oscillators and a threshold nonlinearity so that the frequencies never fall below the overall theta frequency (Fig. 6D, [19]). This model leads to phase precession for runs in any direction. We simulated this model on the entire data set of measured runs in the 2D arena and analyzed how phase-precession slopes depend on properties of the run – just as we did for the in-vivo data. The model, by construction, makes the phase-precession slope independent of the animal's speed (Fig. 6E), which is in agreement with the experimental data for straight runs (Fig. 3F). However, the model also predicts that phase precession should be independent of path length, tortuosity, and eccentricity (Fig. 6F-H). In contrast, the in-vivo data revealed a significant dependence of phase precession on these variables (Fig. 3A, E, G). Furthermore, the model does not show a preferred phase at field entry (p = 0.17, Rayleigh test for circular uniformity), which implies that the phase offset is not constant from run to run. Although some grid fields clearly showed preferred entry phases in the in-vivo data (e.g. Fig. 2B), the overall preference was weak (Fig. 5F, Fig. S17 in File S1), such that this model prediction could not be tested with our data.

## Discussion

Data from rats running on linear tracks provide evidence that the spike phases of mEC grid cells encode spatial distance [11], [18]. This finding extends directly to two-dimensional environments, as was also recently shown by Climer et al. [14] and Jeewajee et al. [13] based on data pooled across multiple runs and firing fields. In addition, our run-by-run analysis revealed a number of unexpected results: spikes precessed at about twice the slope that one estimates from trial-averaged data; the slope of phase precession was the same across all layers of mEC, even for spikes of layer III cells, which were thought not to participate in phase precession [11]; and the sequence of spike phases depended on the properties of the rat's path through a firing field. Long or winding runs through a firing field led to weakest phase precession whereas runs that skirt the edge show steeper rates of phase precession. For this analysis, we measured phase against the total distance traveled within the field, as this distance is easily defined. Based on a more complex measure of the spatial variable and using pooled data, Climer et al. [14] and Jeewajee et al. [13] found that both the (normalized) slope and the correlation of phase precession were the same for runs closer to the edge versus those that passed through the center. These observations are in line with our results because a constant normalized slope implies steeper absolute slopes for the shorter tangential runs. Our run-by-run analysis revealed that the slopes are steeper at a field's periphery (Fig. 3G), despite the fact that both phase range and average path length for these runs were lower. Similar results were found by Huxter et al. [34] (cf. their Fig. S2); in their study on phase precession in CA1 place cells, they introduce a discontinuous measure of space to define phase precession in two-dimensional environments. These authors also noted that phase precession in CA1 depends on the path: for increasingly complicated trajectories, phase precession ceased.

Whereas the phase of hippocampal CA1 neurons for rats running on a linear track correlates more strongly with the distance traveled than with the time elapsed in pooled data [33], at the single-run level in two-dimensional environments, position and time correlated equally well with the phase variable in mEC (Fig. 4). The spike phases exhibit similar single-run position and time-correlations as reported for place cells in the hippocampus [15].

Mizuseki et al. [17] reported that only a minority of cells in mEC display pooled phase precession and that pooled phase precession is layer specific. However, on single runs, the phase *does* precess in many cells across all layers; in fact, the slopes of phase precession are statistically indistinguishable across layers (Fig. 5C). The correlation between phase and position in layer III, however, is weaker, but still more than five times larger than the pooled data suggest (Fig. 5B, 5D). As the phase of the theta rhythm at the time-point of field entry is generally random, one possible explanation is that superimposing data from different runs masked the weakly correlated phase precession in layer III. However, the data set's statistics are too limited to test this hypothesis.

Previous studies also report low rates of spike-phase precession in entorhinal cortex, with a slope of around -3 deg/cm in both Hafting et al. [11] and Mizuseki et al. [17]. This rate stands in stark contrast to the -10 deg/cm slope based on single runs drawn from the same data as in Hafting et al. [11] that we describe here (Fig. 2F). Part of the discrepancy can be explained by the difference between single runs and pooled data (Fig. 2E); while another part can be explained by the quantification of phase precession: Kempter et al. [28] showed that linear-linear regression tends to underestimate the slope and range of phase precession compared to circular-linear regression, as we have used. The slope of phase precession in mEC is in line with the phase-precession slope observed in hippocampus (−7.6 deg/cm on average) and medial prefrontal cortex (−9.4 deg/cm on average, both numbers from [35]). Jeewajee et al. [13] demonstrated pooled phase-precession in open environments in both hippocampal place cells and entorhinal grid cells, although the proportion of significantly phase-precessing cells was higher in hippocampus than in entorhinal cortex. In this study, the authors mapped individual runs to the unit circle, normalized running direction and pooled these normalized runs, which allows for a clear visualization of phase precession in open environments. However, using this procedure, it is rather complicated to investigate the impact of the animal's trajectory on phase precession as the properties of individual runs are lost by normalization and pooling.

Hafting et al. [11] found the average phase range – in pooled data – to be about 165°, which is in broad agreement with the single-run phase ranges in our analysis (171° for runs <30 cm) and in hippocampus (180°) [15]. Phase precession, therefore, exhibits consistent characteristics throughout the entorhinal-hippocampal loop [12], [34].

Which cells, therefore, drive phase precession? Even within the same layer of mEC, cellular biophysics and morphology vary [36]–[38]. Stellate cells in mEC are thought to support phase precession, as they display subthreshold resonance and membrane potential oscillations in the theta frequency range [39], [40], which can also affect spike patterns [41]–[43]. On the other hand, pyramidal cells in layer III act as low-pass filters (at least at the soma). Interlaminar connections [44] could allow one layer to impose its phase precession onto another layer, or at least influence that layer. Likewise, the strong phase precession of principal cells in the hippocampal CA1 subregion may – at least in part – stem from single-run phase precession in mEC [45], [46].

We found that the observed phenomena in mEC were not in agreement with oscillatory interference models [19], [20], [32], which explain both grid-cell formation and phase precession as the result of a common mechanism, namely the interference of oscillations at different frequencies. In such models, grid spacing and rate of phase precession are linked. Pure phase precession, as opposed to a mixture of phase precession and recession, required nonlinear rectification and the involvement of six oscillators, instead of three. While this oscillatory interference model is still consistent with omnidirectional and speed-independent phase precession, it did not reproduce the features of biological phase precession derived from the single-run analysis: the model displayed a constant rate of phase precession, regardless of the path taken. However, it seems that including a “somatic” baseline oscillation in the model would lead to steeper phase-precession slopes at the edges of a firing field [14]. Extensions of continuous-attractor networks that include mechanisms for spiking and oscillation [22], [24] inherit phase precession from the oscillatory interference model and generate realistic grid spacings and rates of phase precession. However, the phase precession remains independent of the properties of the path, which is inconsistent with the experimental observations. Navratilova et al. [24] suggest that a conjunction of intrinsic and extrinsic mechanisms could generate different slopes of phase precession on field entry and field exit; but such a model cannot generate different rates of phase precession for tangential and central paths through a grid field. Therefore, it remains an open challenge to find a minimal model that accurately describes entorhinal phase precession in two-dimensional environments.

## Supporting Information

File S1 **Contains 18 additional figures.**
(PDF)
