# Supplementary material for: Movement Dependence and Layer Specificity of Entorhinal Phase Precession in Two-Dimensional Environments
Source: PLoS One. 2014 Jun 24;9(6):e100638. doi: 10.1371/journal.pone.0100638 (PMC4069107; doi:10.1371/journal.pone.0100638)
Supplement: File S1 — Contains 18 additional figures. (PDF) [file pone.0100638.s001.pdf]

Supporting information

“Movement dependence and layer specificity of entorhinal phase precession in two-dimensional environments”

Eric Reifenstein, Martin Stemmler, Andreas Herz, Richard Kempter, Susanne Schreiber

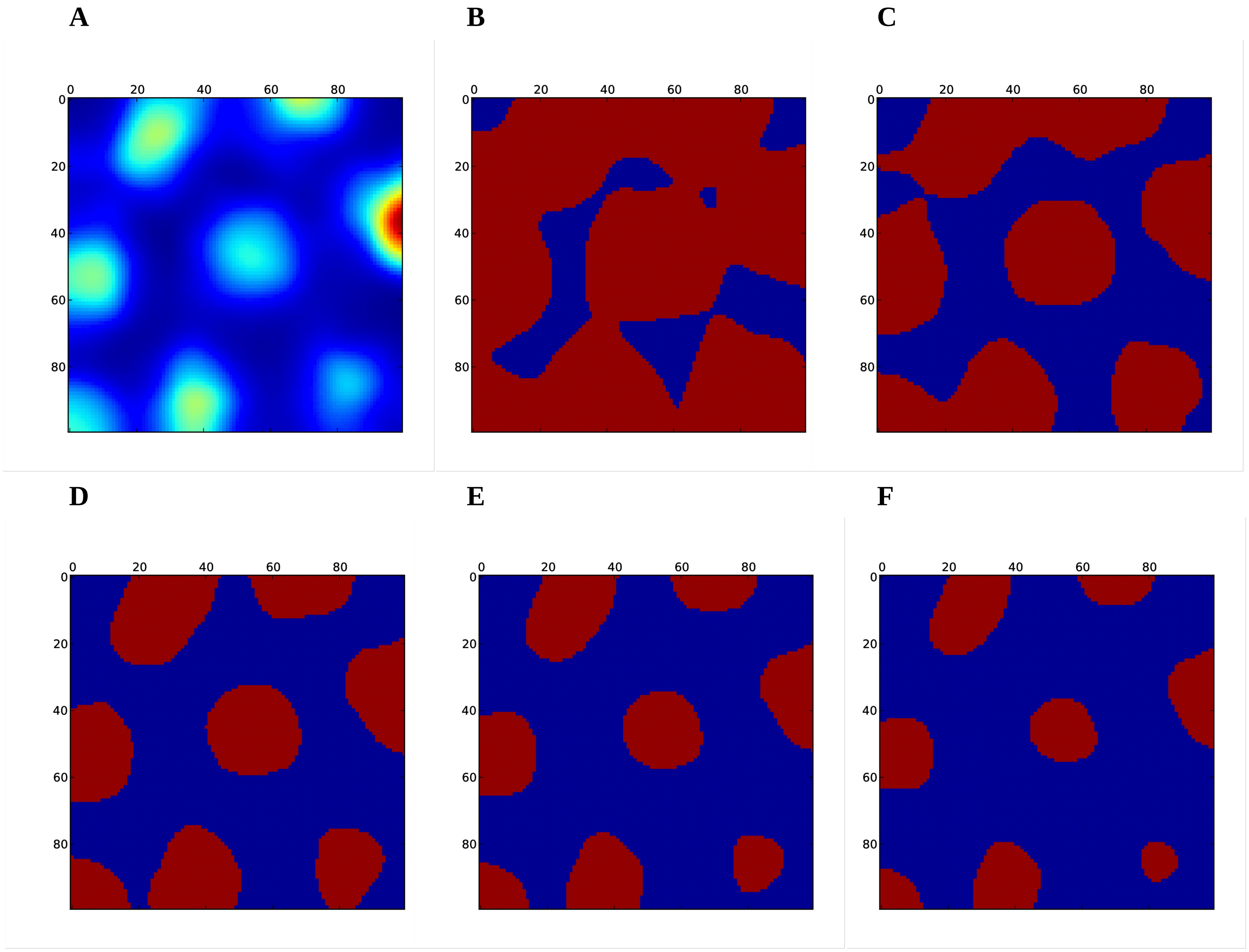

Figure S1: (A) Firing-rate map of a grid cell. (B)-(F) Different thresholds for the selection of firing fields (B: 5%, C: 10%, D: 15%, E: 20%, F: 25% of the peak firing rate). Too low thresholds led to merged fields, whereas too high values excluded weak fields from the analysis. We hence chose a threshold of 20% for all the analysis in the manuscript. The preliminary fields that were found by this criterion were then extended to 20% of the individual field's peak firing rate. For comparison we note that in hippocampal data, where firing rates are generally higher than in our data and often only a single firing field is present, frequent choices for threshold values are 5% or 10%.

A **B**

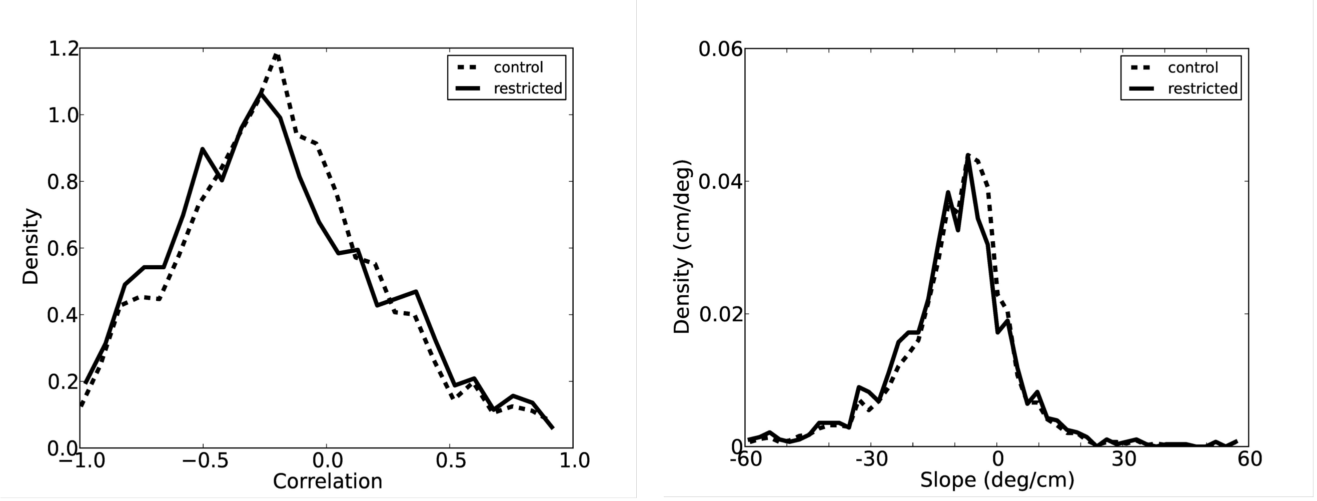

Figure S2: We tested two additional criteria for the selection of runs: a single run must not be shorter in duration than three theta cycles and the rat must not move slower than 1 cm/s during any time of the field traversal. The distribution of phase precession correlation (A) and slope (B) were affected only marginally; changes were not statistically significant (correlation: Wilcoxon test p=0.10, slope: t-test p=0.07).

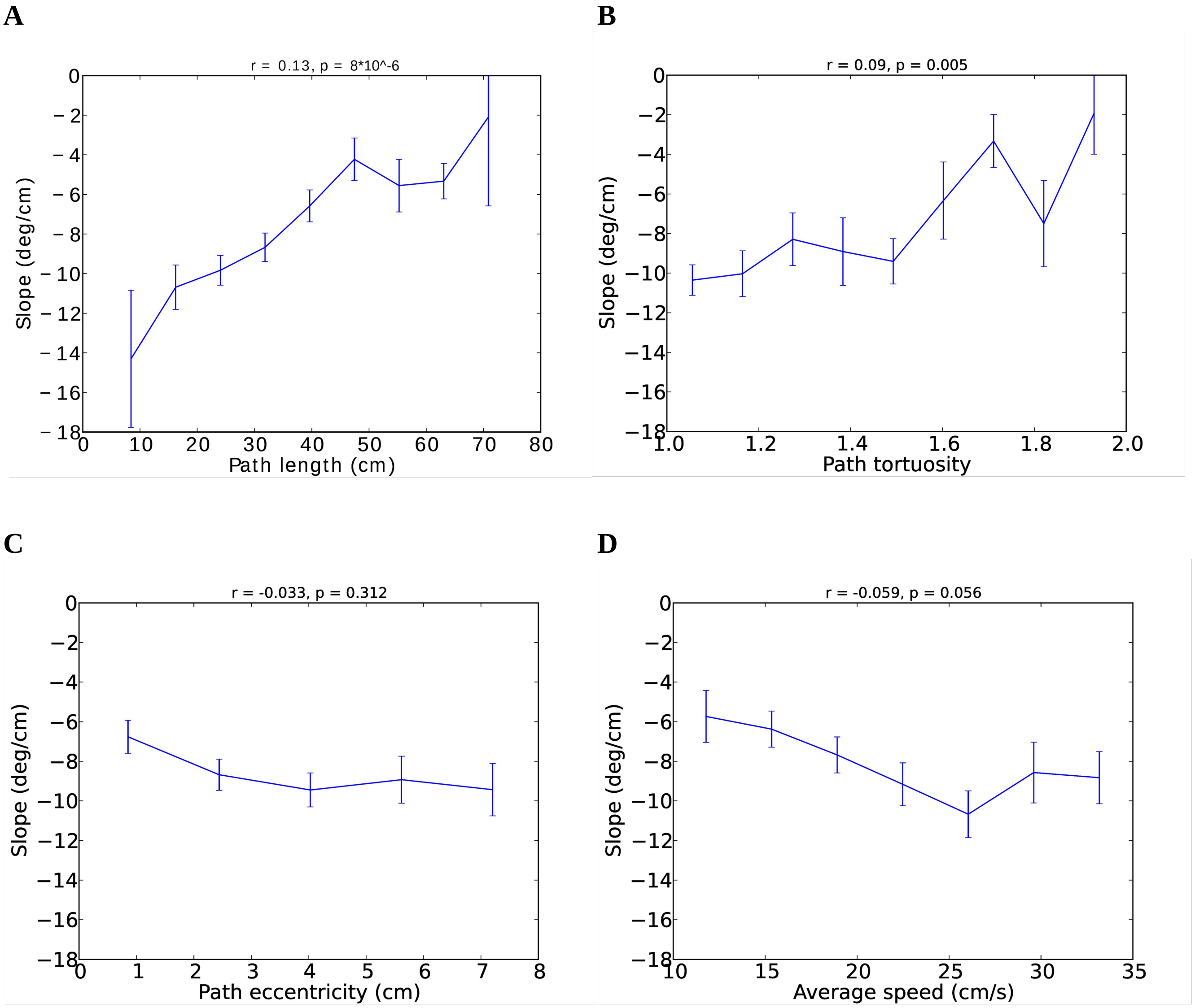

Figure S3: Using the same additional restrictions for selecting runs as in Fig. S2, i.e. runs must not be shorter than three theta cycles and speed is always larger than 1 cm/s, we could reproduce the effects of path length (A) and tortuosity (B). The restrictions reduced the number of runs included in the analysis (in particular those with lower speed), so that the dependence of phase precession slope on eccentricity (C) and running speed (D) did not meet the criterion for statistical significance anymore.

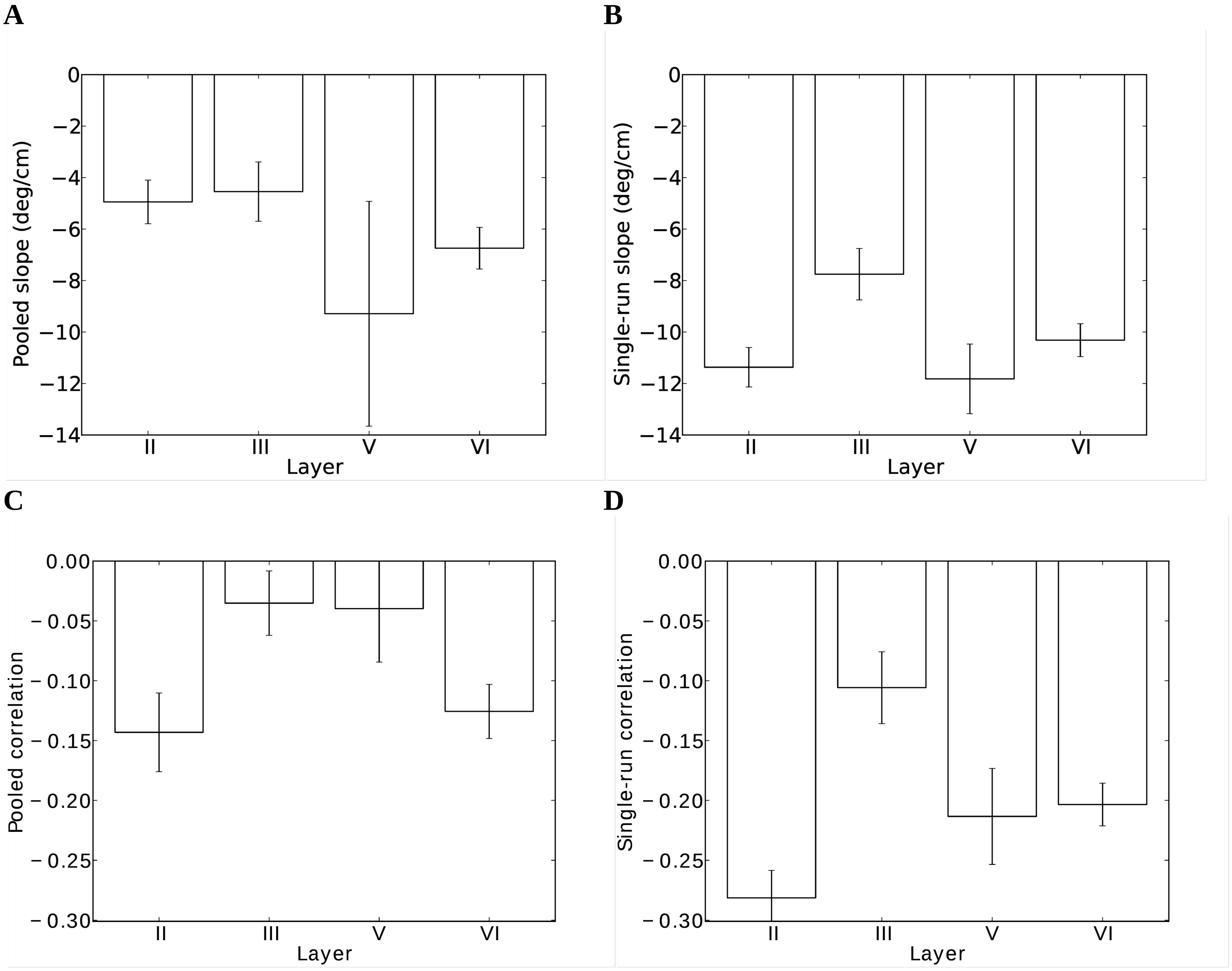

Figure S4: Layer specificity of phase precession for a stricter selection of runs. When the analysis is restricted to runs where the animal did not move slower than 1 cm/s, the results for pooled correlation (C) and single-run correlation (D) are well preserved. Interestingly, the difference in pooled slopes between layers II and III is reduced for the stricter selection of runs (A), whereas the difference in single-run slopes between layers II and III is increased (B).

A

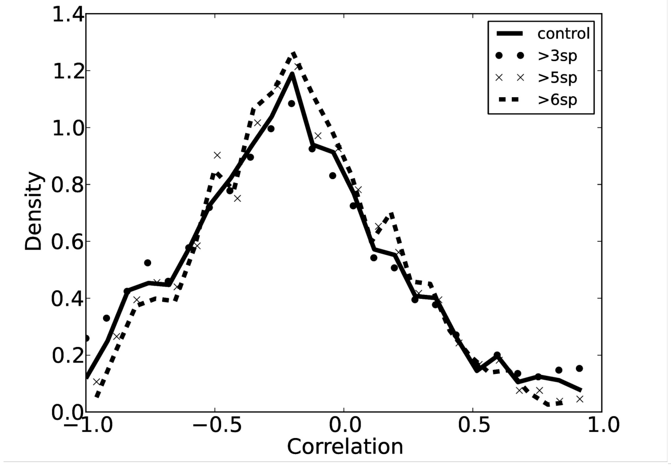

B

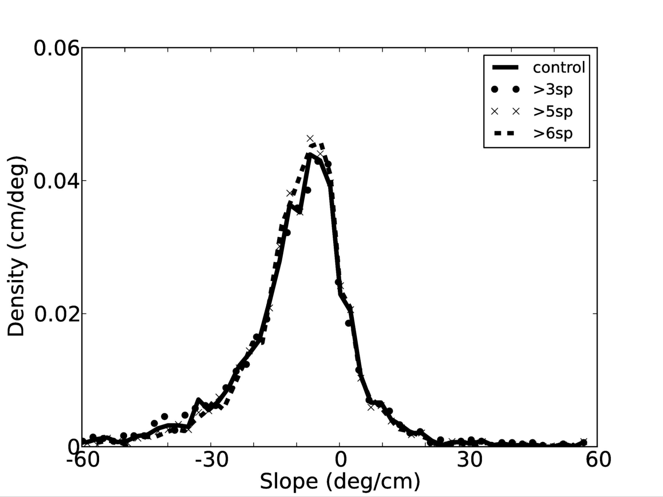

Figure S5: Throughout the manuscript, only runs with more than four spikes were included in the analysis (here termed control condition). To exclude a strong dependence of the results on this selection criterion, we also estimated phase precession correlation (A) and slope (B) for runs with more than 3, 5, or 6 spikes, respectively. Phase-precession estimates were only mildly affected by these different criteria.

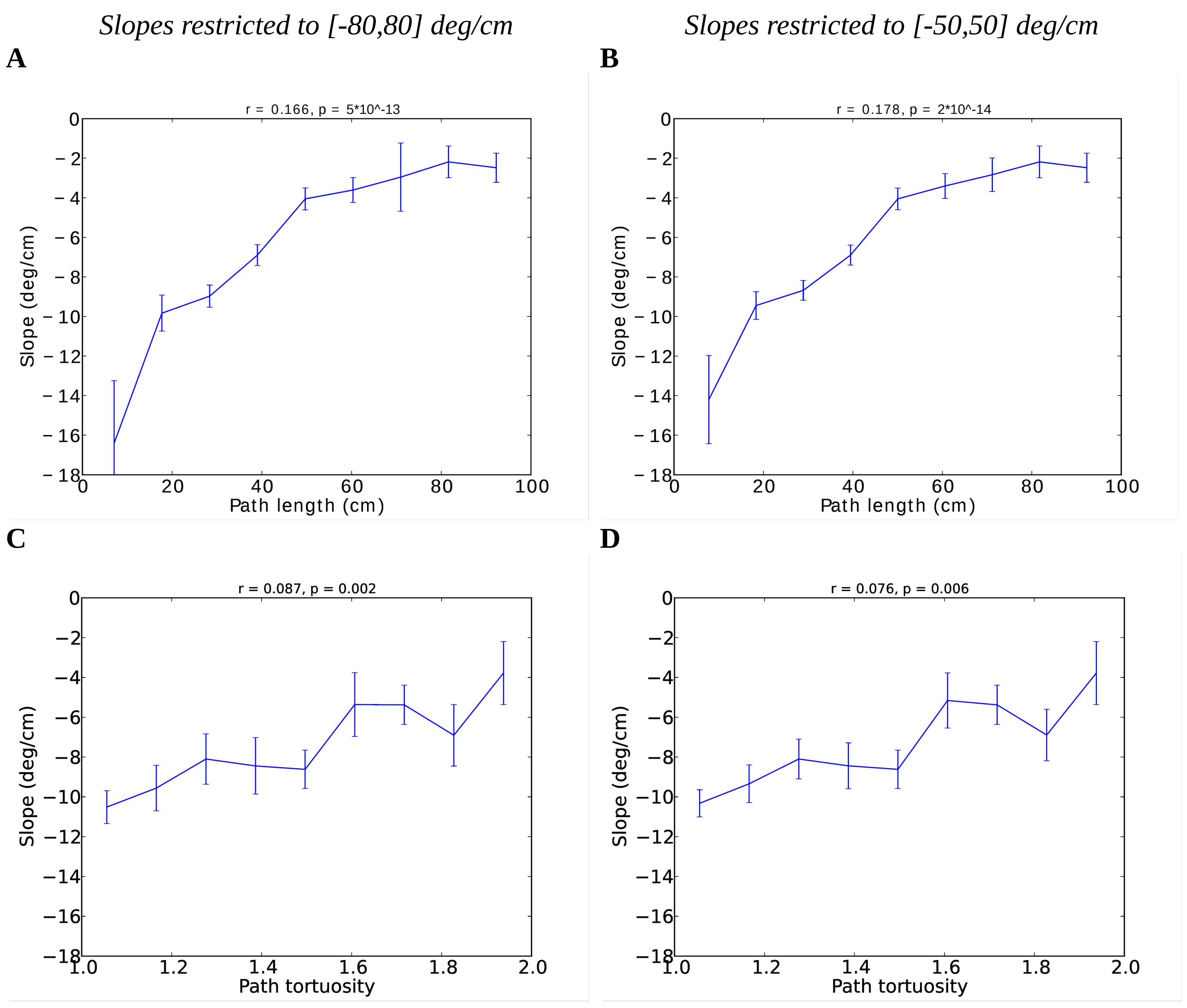

Slopes restricted to [-80,80] deg/cm
Slopes restricted to [-50,50] deg/cm
A
r = 0.166, p = 5*10^-13
Slope (deg/cm)
Path length (cm)
B
r = 0.178, p = 2*10^-14
Slope (deg/cm)
Path length (cm)
C
r = 0.087, p = 0.002
Slope (deg/cm)
Path tortuosity
D
r = 0.076, p = 0.006
Slope (deg/cm)
Path tortuosity

Slopes restricted to [-80,80] deg/cm *Slopes restricted to [-50,50] deg/cm*

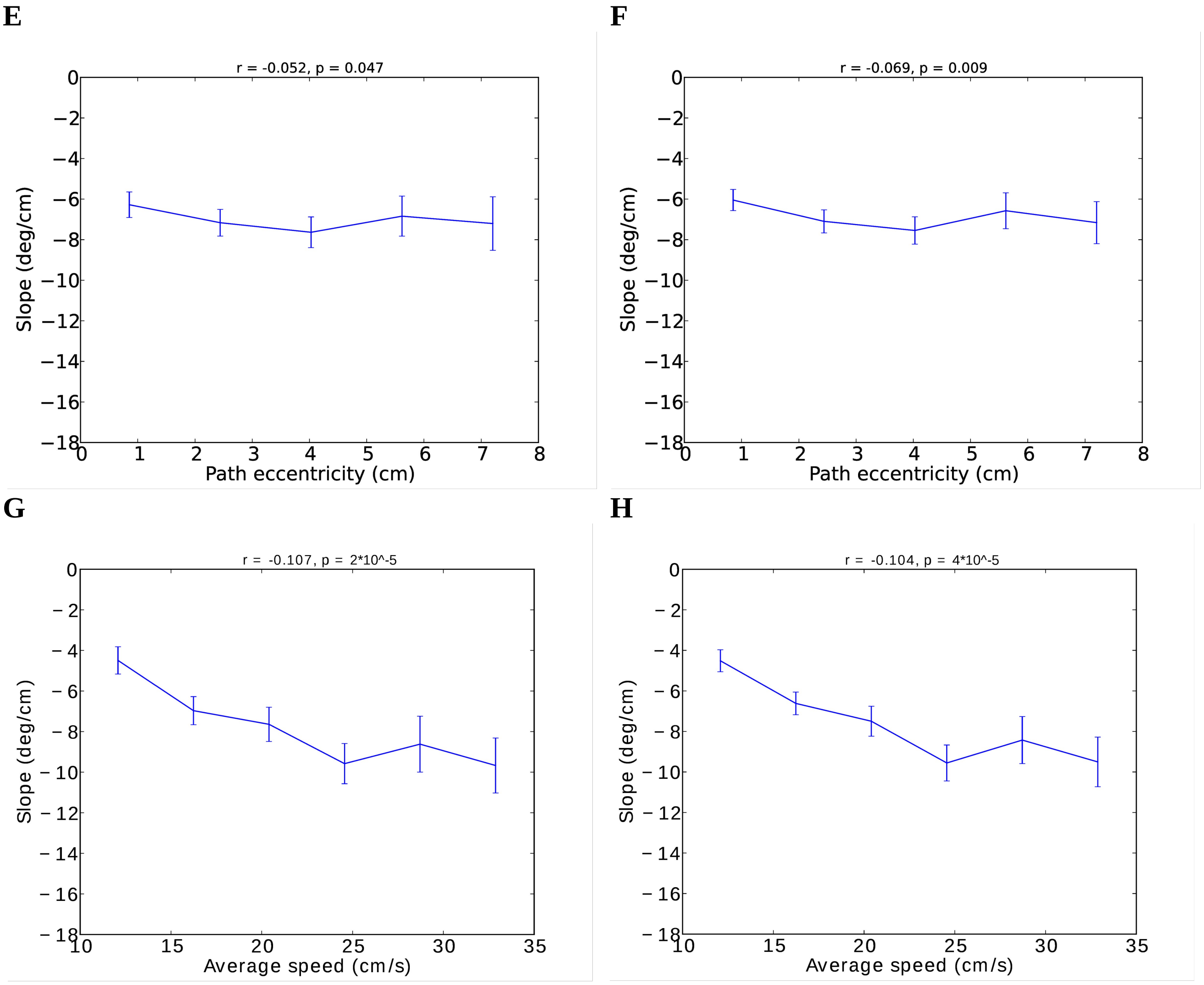

Figure S6: Single-run slopes were restricted to the range [-60,60] deg/cm throughout the manuscript. The dependence of phase precession on the properties of the path through a firing field can be reproduced when this restriction is relaxed, so that the slope is required to lie within the broader range of [-80,80] deg/cm (A, C, E,G), or tightened, so that the slope lies within [-50,50] deg/cm (B, D, F, H); compare to Fig. 3 in the manuscript.

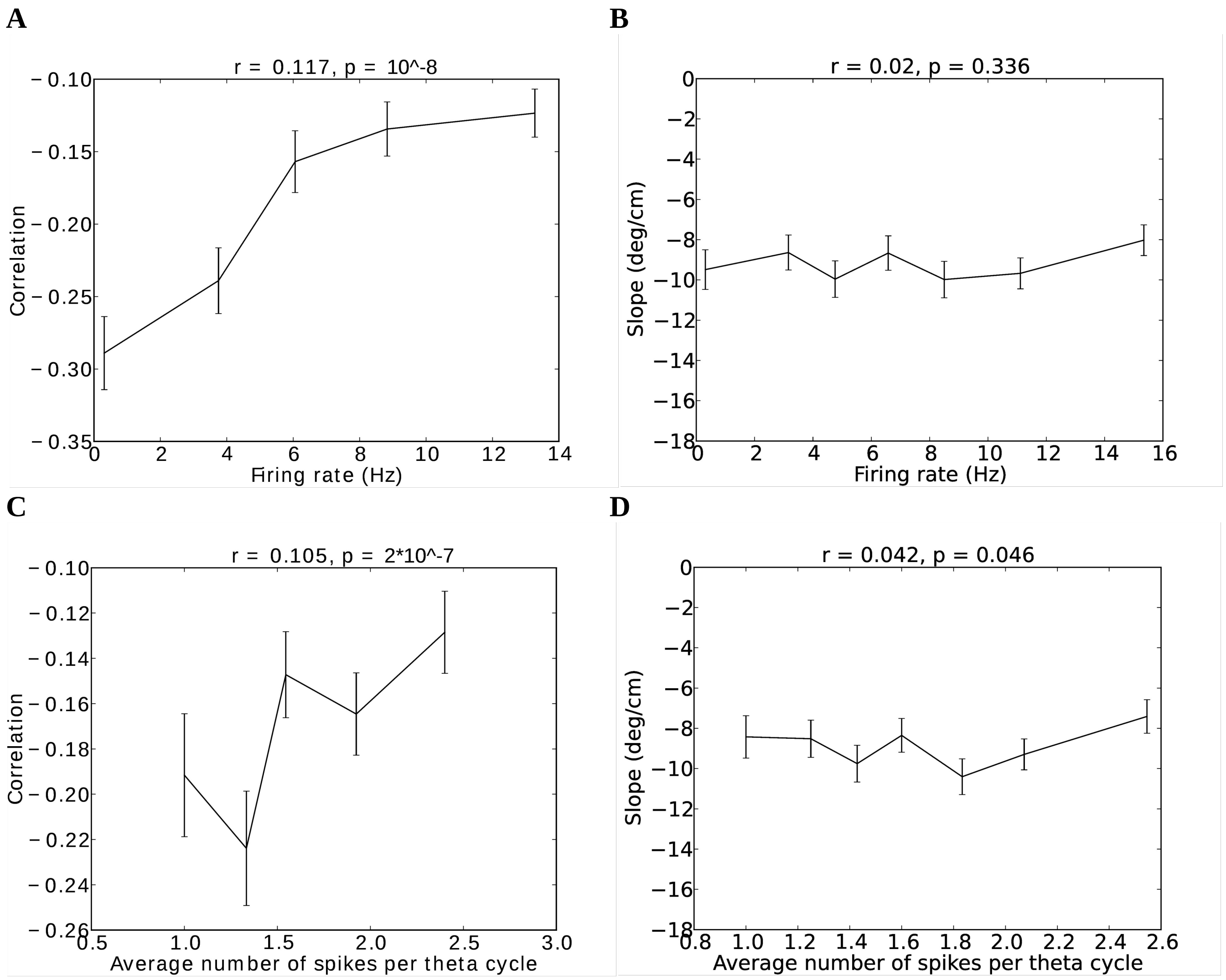

Figure S7: How does firing rate influence phase precession? Here we show the dependence of phase precession correlation (left column) and slope (right column) on firing rate using two estimates: the number of spikes divided by run duration (i.e. average firing rate; top row) and the average number of spikes per theta cycle in a run (bottom row). Firing activity and phase-precession correlation were weakly correlated (A and C), whereas slope was not affected by firing rate (B) or average number of spikes per theta cycle (D).

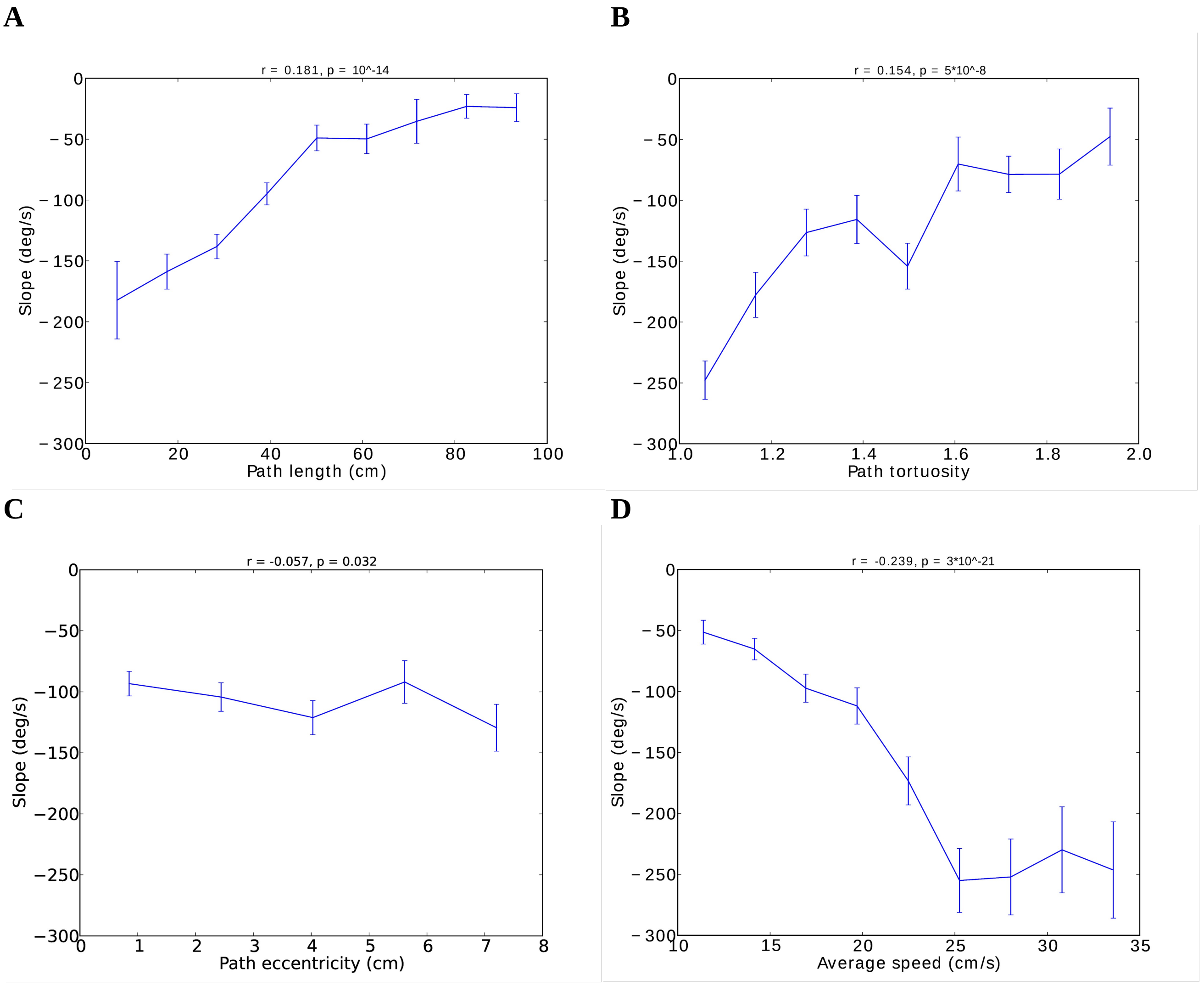

Figure S8: Salient features of the animal's path through a grid field affect the phase-time slope. For the phase-time slope, the path-length effect (A), the tortuosity effect (B) and the eccentricity effect (C) could be reproduced (see Fig. 3). (D) The phase-time slope correlated negatively with the speed of the animal – this is expected as there was no or only weak correlation of the phase-position slope with speed (Fig. 3F).

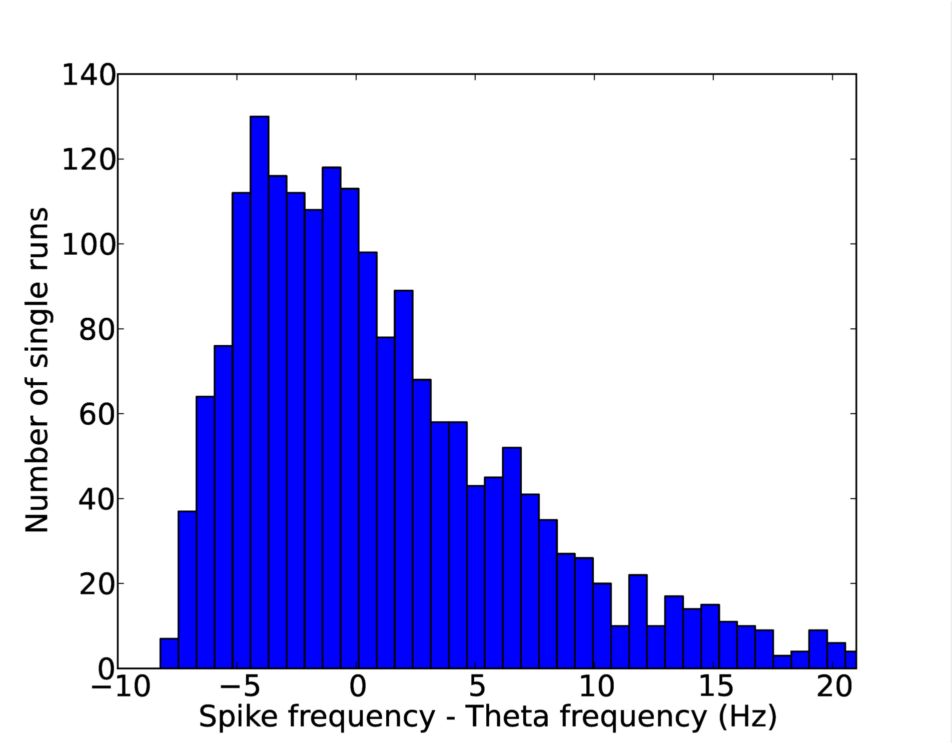

Figure S9: To investigate phase precession, previous studies measured the difference of the oscillation frequency of neuronal firing and the LFP's theta rhythm (Mizuseki et al., 2009). There, phase precession manifests itself as a positive frequency difference. In our single-run analysis, however, the difference was negative in many single runs (median difference -0.15 Hz) because cells often skipped theta cycles without spiking. Therefore, the frequency difference is not a suitable measure for assessing phase precession in entorhinal grid cells.

A

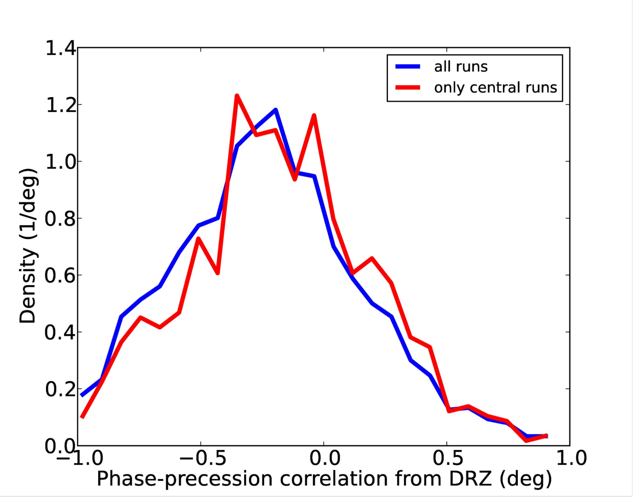

B

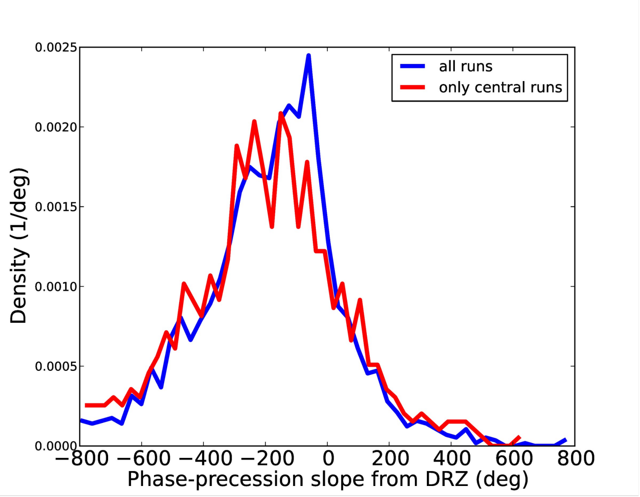

Figure S10: The so-called Directional Rate Zone measure (DRZ) was introduced by Huxter et al. (2008). Here, we show that phase precession was also evident with respect to the DRZ measure in our data for both correlation (A, -0.19 [-0.22, -0.18] for all runs, -0.15 [-0.17, -0.11] for central runs, median correlation ± 95% confidence interval) and slope (B; -164° ± 5° for all runs; -180° ± 9° for central runs, mean slope ± s.e.m.). Runs with an eccentricity of smaller than 3 cm were defined as central.

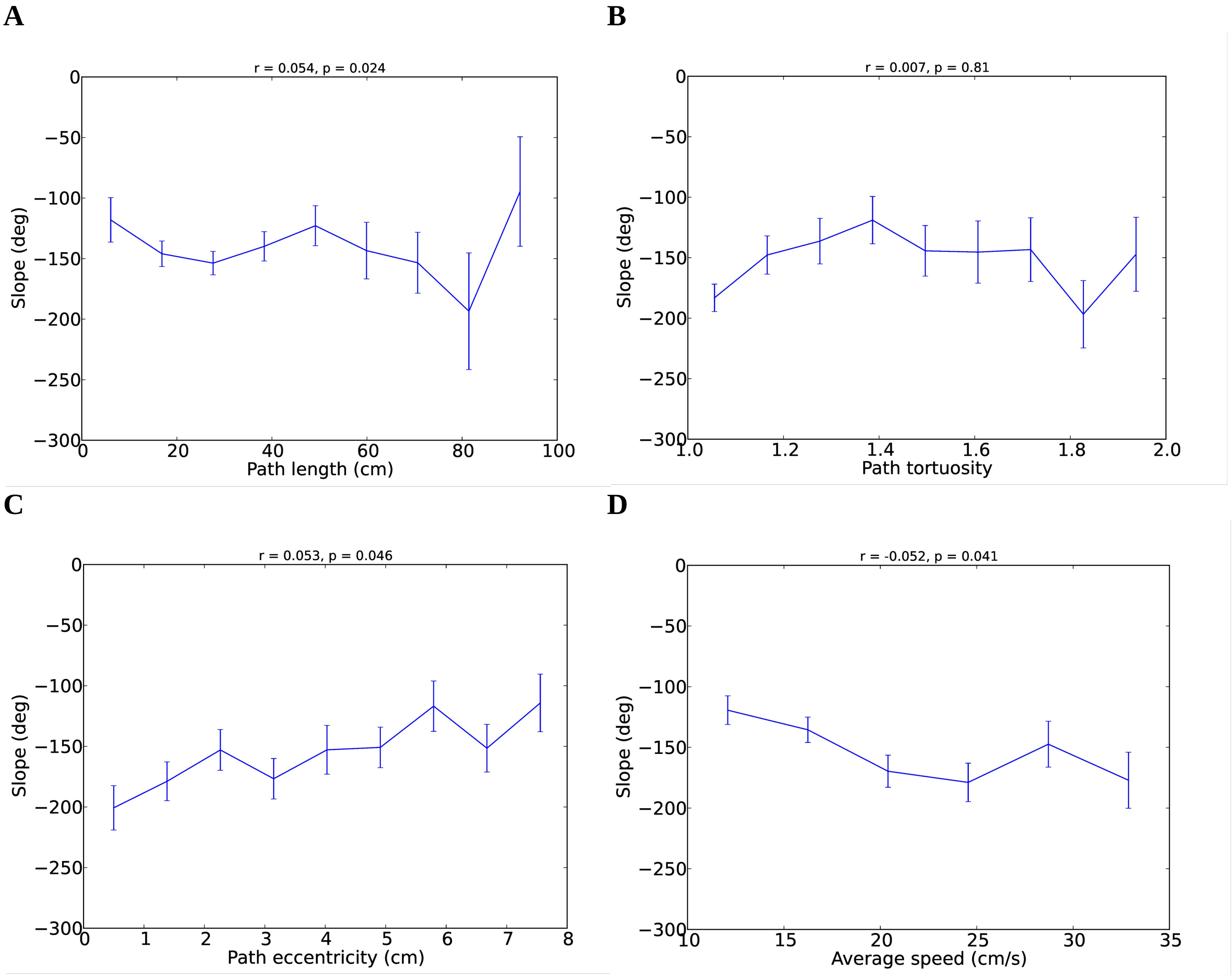

Figure S11: Although the DRZ measure can be used to show phase precession in our data, it does not capture the dependence of phase precession on the movement statistics during field traversals. The reason lies in the discontinuity of the measure along peripheral trajectories (i.e. trajectories that do not pass a field's center). For these runs, the absolute values of the measure are always non-zero and the sign inverts instantly when the animal changes from running 'towards' the center to running 'away' from it. This discontinuity distorted the estimate of phase-precession slope and weakened the effects of path length (A), tortuosity (B), or speed (D) on the phase-precession slope (from DRZ). The effect of eccentricity (C) was even inverted. The farther the path lies from the center of the firing field, the shallower the regression line between phase and DRZ became.

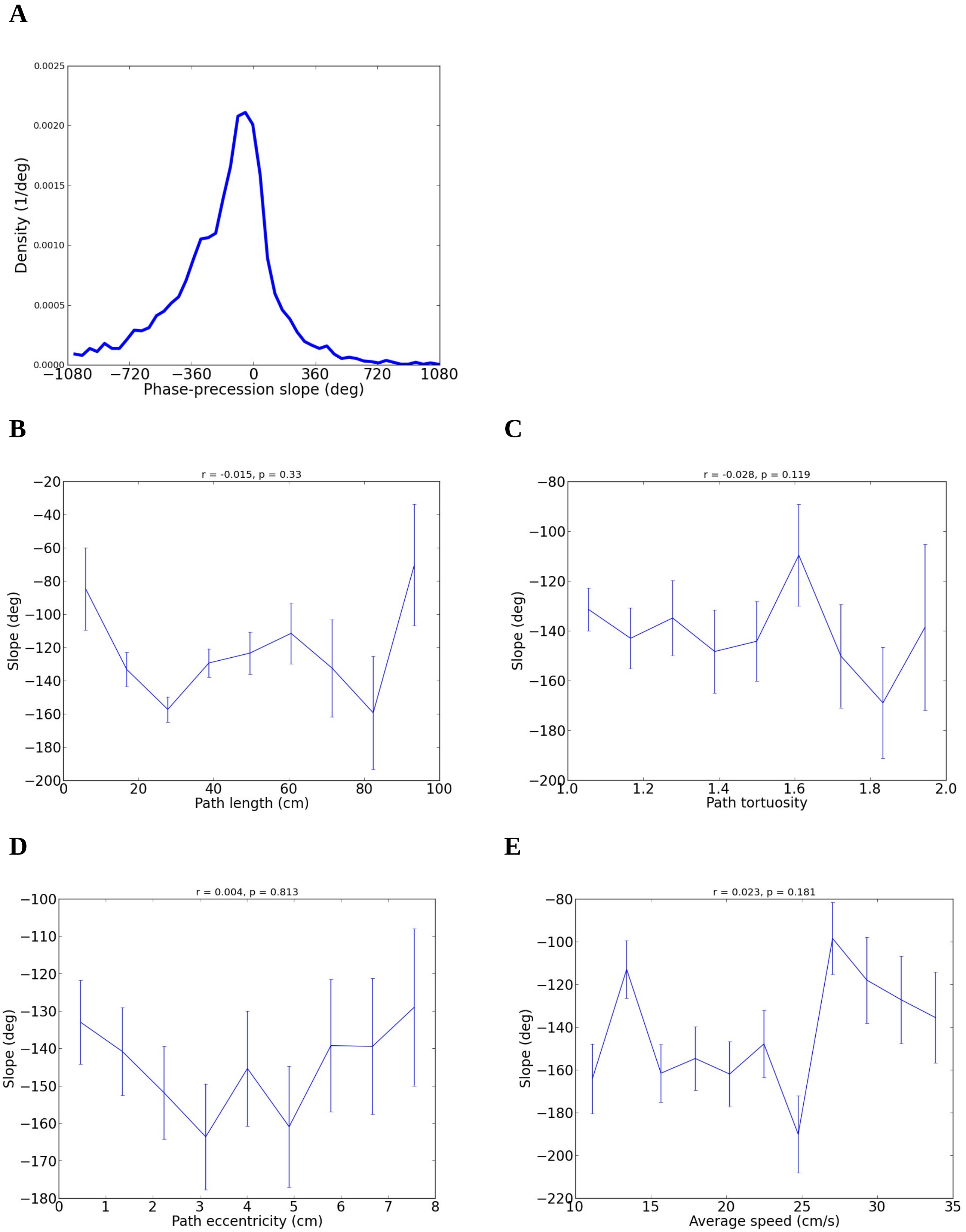

Figure S12: Single-run slopes calculated from normalized running distance do not depend linearly on the properties of the rat's trajectory. (A) Distribution of phase-precession slopes. Distance is normalized within each run, ranging from 0 (at field entry) to 1 (at field exit). (B) to (E) Properties of the rat's path are not significantly correlated with the slope of theta phase vs. normalized distance.

A **B**

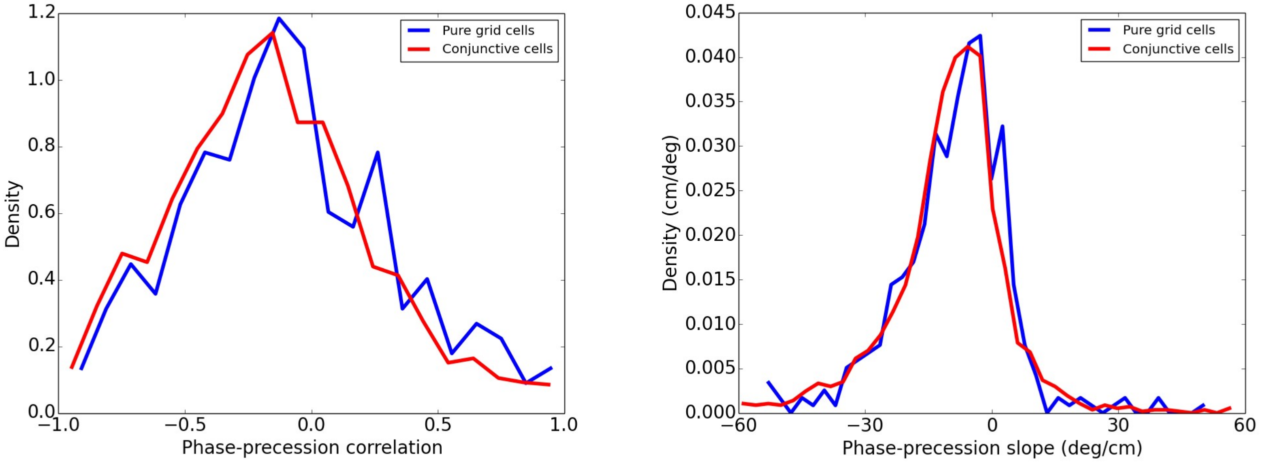

Figure S13: Conjunctive cells exhibit phase precession. (A) Phase-precession correlation in conjunctive cells appears to be slightly more negative compared to pure grid cells (p<0.001, Mann-Whitney rank test). (B) The slopes of pure grid cells and conjunctive cells are not significantly different. If at all, conjunctive cells exhibit slightly more negative slopes.

A **B**

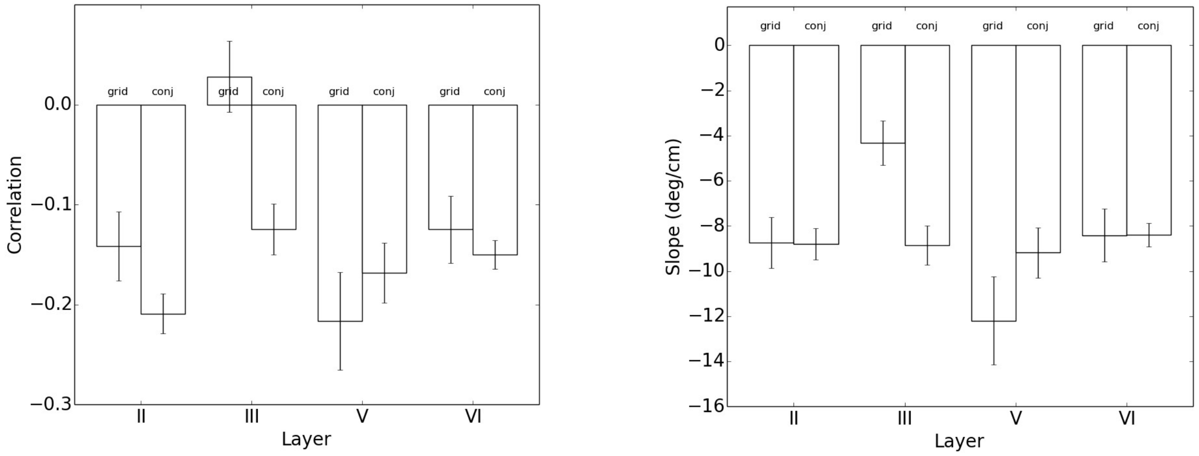

Figure S14: (A) Conjunctive cells in layer III exhibit clear phase precession whereas pure grid cells do not. Also in layer II, phase-precession correlation is tighter in conjunctive cells than in pure grid cells (layer II: p=0.048, layer III: p<0.001). (B) Phase-precession slopes in layer III are significantly steeper in conjunctive cells as compared to pure grid cells (p=0.002, t-test). For the slopes in layers II, V and VI no statistical differences could be found.

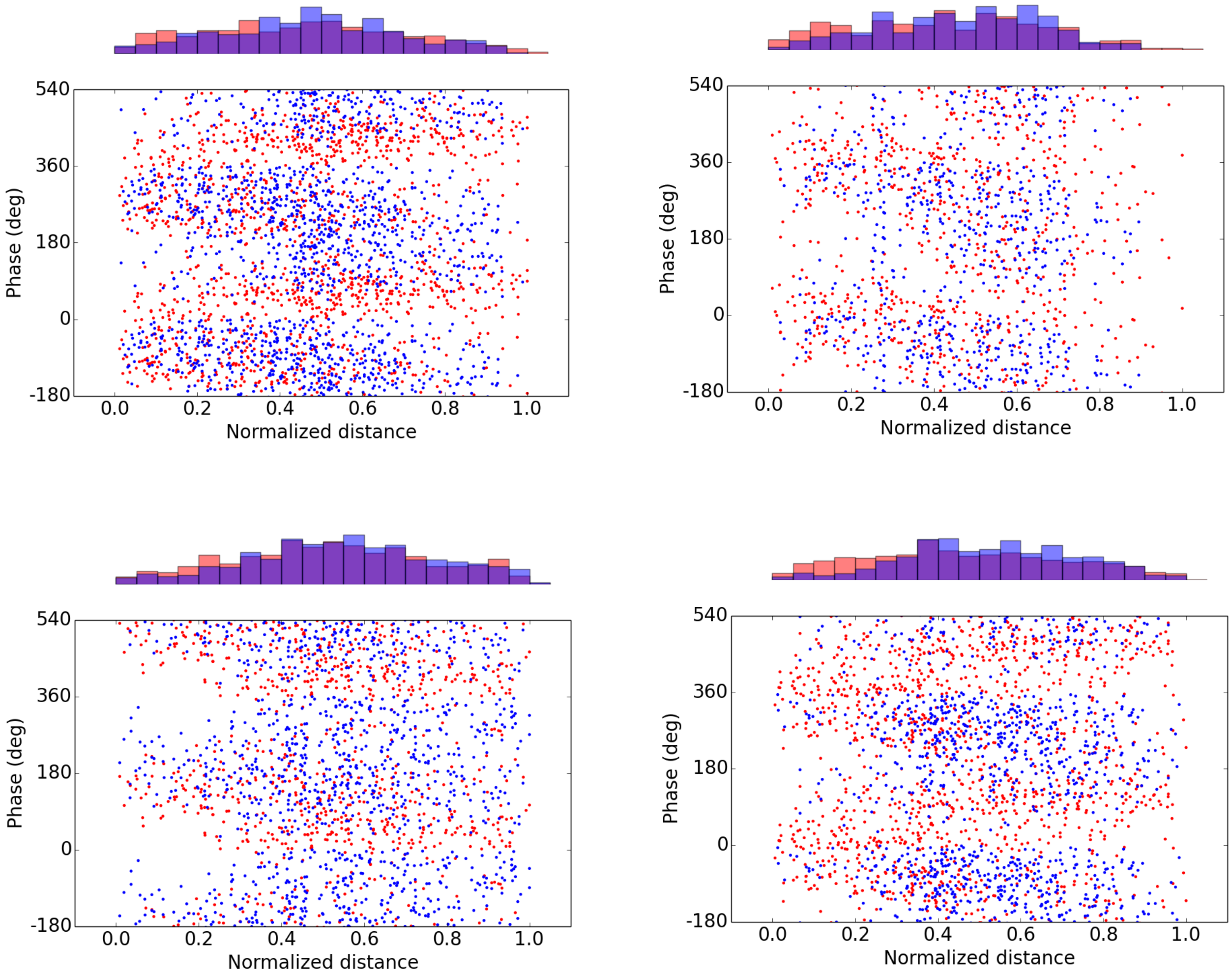

Figure S15: Burst firing is commonly observed in grid cells. Examples of pooled-data phase precession for four grid fields of different grid cells (the first three fields are the same as in Fig. 2B). Spikes within a theta cycle were separated in leading spike (the first spike in the cycle) and follower spikes (all spikes later in the cycle). Leading spikes are shown in red, follower spikes in blue. Histograms show the corresponding distributions of normalized distances (averaged across phases). Climer et al. (2013) found an increased portion of follower spikes towards the end of the run for linear-track data. In open environments, we find examples of grid fields that also show this behavior. For pooling, the distance is normalized by the total path length in each individual run.

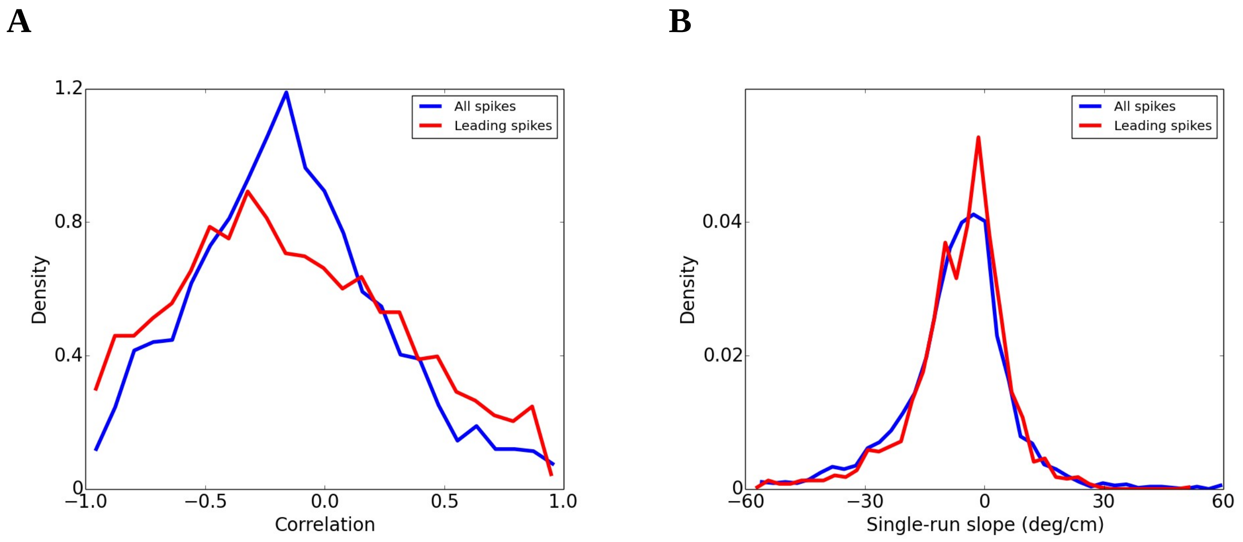

Figure S16: In open environments phase precession remains when the analysis is restricted to the leading spikes only. (A) Distribution of single-run correlation for all spikes and leading spikes. Leading spikes tend to result in a slightly tighter correlation of phase and distance, but this effect is statistically not significant. (B) Distribution of single-run slopes for all spikes and leading spikes. No statistical difference could be found.

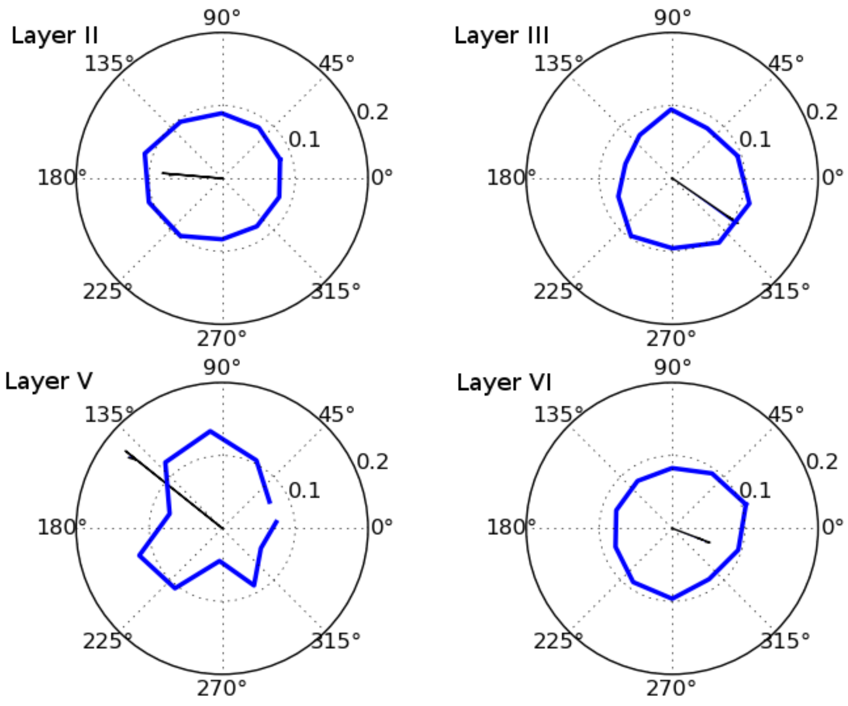

Figure S17: Single-run offsets from the circular-linear regression between travelled distance and theta phase. Layers II, III and VI show a non-uniform angular distribution of offsets (Rayleigh-test: p = 0.015, p = 0.0005, p = 0.035, respectively). For layer V (p = 0.2) only a limited amount of data was available. Although the distributions are mostly non-uniform, the vector strength is generally low (black lines), indicating large phase jitter. This might explain why the preferred offsets differ from the preferred phase of the first spike (Fig. 5F).

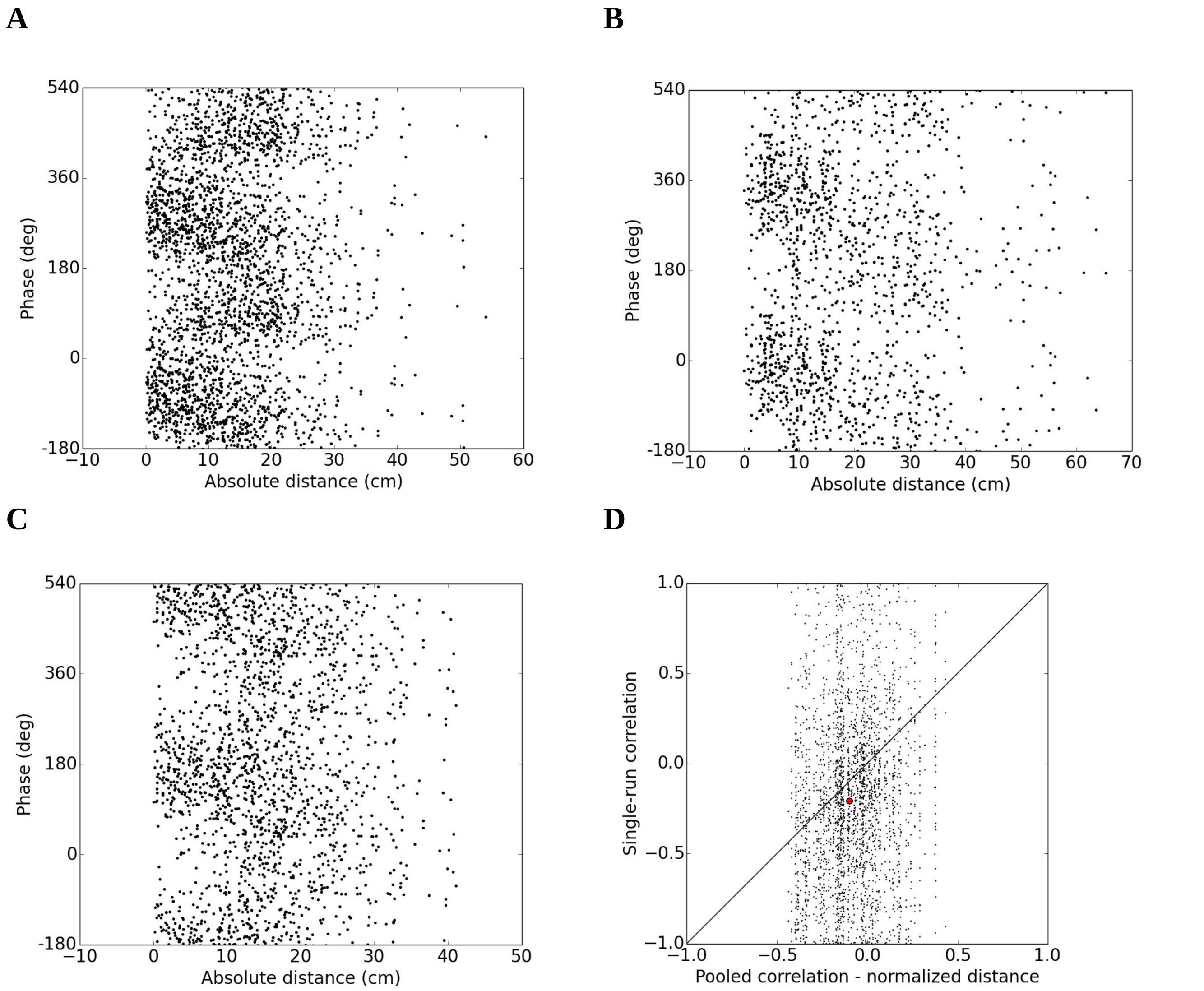

Figure S18: (A) – (C) Examples of pooled phase precession with respect to absolute distance along the path. Data represent the same three grid fields as in Figure 2B. Phase precession is also evident with respect to the absolute distance, though normalized distance seems to be the better regressor for theta phase. In the main text, we compared single-run correlation and pooled correlation, both calculated using the absolute distance. (D) reproduces this finding (Figure 2E) for pooled correlation that is calculated from the normalized distance. Phase-precession correlation is significantly stronger in single runs than in pooled data from normalized runs (p<10^-10, Mann-Whitney rank test). The red dot indicates medians of pooled correlation and single-run correlation.
